# Engineered Probiotic‐Powered Micro‐Rod Robot Bomb for Thrombus‐Penetrating Explosion

**DOI:** 10.1002/advs.202501304

**Published:** 2025-08-28

**Authors:** Riyue Liu, Jiale Chen, Yuanyuan Li, Nawsherwan, Cuilian Dai, Yan Wang, Jinrong Zheng, Kecheng Zhou, Mangmang Sang

**Affiliations:** ^1^ Xiamen Cardiovascular Hospital of Xiamen University, School of Medicine, Fujian Branch of National Clinical Research Center for Cardiovascular Diseases Xiamen 361006 China; ^2^ The Second Affiliated Hospital and Yuying Children's Hospital of Wenzhou Medical University, School of Rehabilitation Medicine Wenzhou Medical University Wenzhou Zhejiang 325035 China; ^3^ Hebei Key Laboratory of Cardiac Injury Repair Mechanism Study The First Hospital of Hebei Medical University Shijiazhuang 050031 China

**Keywords:** micro‐rod, penetrating, probiotic, robot, secondary thrombosis

## Abstract

Conventional thrombolytic agents demonstrated limited efficacy in treating thrombotic disorders characterized by narrow therapeutic windows and progressive vascular injury, lacking the required precision, timeliness, and treatment durability. Here an engineered probiotic powered micro‐rod robot for targeted and penetrative treatment for thrombus is developed. This micro‐rod robot (_Sr_EcN_PL_) using natural probiotics as bio‐carriers, functionalized with thrombolytic carbon nanotubes and platelet membrane‐coated nanoparticles loaded with targeted vasodilators. The robot exploits the rapid movement of probiotics, combined with the active targeting ability of the platelet membrane, to achieve rapid and precise drug delivery to thrombus tissue. Based on the natural rod‐shaped structure of probiotics, _Sr_EcN_PL_ can deeply penetrate thrombus tissue, achieving faster thrombolysis efficiency. The thrombolytic micro‐robot combines thrombus‐targeting capability with prolonged circulation time and controlled vasodilation, maintaining vascular patency and inhibiting secondary thrombus. Results indicate that this micro‐robot can quickly and accurately target and penetrate thrombus tissue, extending the biological half‐life of thrombolytic drugs by ≈332 times and enabling sustained thrombolysis. This novel dual‐pronged combined thrombolytic therapy has significant scientific implications for treating thrombotic diseases with narrow therapeutic windows caused by vascular injury.

## Graphical abstract

For thrombotic diseases with narrow therapeutic windows and persistent vascular damage; existing thrombolytic drugs are difficult to achieve precise; timely; and sustained thrombolytic effects. Here an engineered probiotic driven micro‐rod robot for targeted and penetrative treatment for thrombus is developed. This micro‐robot can rapidly and accurately target and penetrate thrombus tissue; significantly prolonging the biological half‐life of thrombolytic drugs (≈332 times) and achieving sustained thrombolysis. This novel dual pronged thrombolytic therapy holds great scientific promise for the treatment of thrombotic diseases with shorter treatment time windows caused by vascular injury.

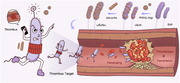

## 1 Introduction

Thrombus constitutes the underlying pathological basis for three major fatal cardiovascular and cerebrovascular diseases: myocardial infarction, stroke, and venous thromboembolism.^[^ ^1^, ^2^ ^]^ Thrombolytic therapy remains the primary clinical intervention for thrombotic diseases.^[^ ^3^, ^4^ ^]^ However, due to poor targeting and short half‐life,^[^ ^5^, ^6^, ^7^ ^]^ commonly used thrombolytic drugs necessitate continuous administration, which can lead to high‐risk adverse bleeding complications.^[^ ^8^, ^9^, ^10^ ^]^ For thrombotic diseases characterized by a narrow therapeutic window and persistent vascular injury, existing thrombolytic drugs struggle to achieve timely and sustained thrombolytic effects.^[^ ^11^, ^12^, ^13^, ^14^, ^15^ ^]^ Develop strategies to enable rapid delivery of thrombolytic drugs to localized thrombotic sites while sustaining prolonged therapeutic action represents a critical unmet clinical need. Such approaches could mitigate hemorrhagic side effects in the circulatory system and prevent secondary thrombus formation.

Over the past decade, advancements in drug delivery technology have reduced the hemorrhagic risks associated with the clinical use of potent thrombolytic agents.^[^ ^14^, ^16^, ^17^ ^]^ Compared with conventional drug delivery systems, biofilm‐biomimetic carrier masking technology offers better biocompatibility and retains the complex functions of membrane donor cells under physiological or pathological conditions.^[^ ^18^, ^19^, ^20^ ^]^ Research has demonstrated that under pathological conditions, platelets actively contribute to thrombus formation while exhibiting intrinsic thrombus‐targeting capabilities. Researchers have improved thrombus targeting and prolonged systemic circulation time by encapsulating thrombolytic drugs in platelet membranes.^[^ ^8^, ^17^, ^21^ ^]^ The research group previously designed neutrophil‐based thrombolytic drug carriers for thrombus treatment.^[^ ^22^ ^]^ However, biofilm loaded drug nanoparticles mainly rely on chemokine stimulation, blood flow, and passive diffusion to reach the lesion site, which may limit their transport speed and therapeutic efficacy as drug carriers.

To improve the mobility of drug delivery carriers, scholars have recently focused on biological micro‐nano robots. The results show that biological micro‐nano robots have extremely high application value in improving drug carrier targeting speed, drug delivery efficiency, and deep penetration. Compared with spherical micro‐nano robots, rod‐shaped robots generate torque, rotation, and rolling phenomena in blood circulation, thereby enhancing their interaction with vessel wall thrombus.^[^ ^14^, ^23^, ^24^, ^25^ ^]^ Rod‐shaped robots also have a lower macrophage uptake rate and longer blood circulation time than spherical particles, which helps improve drug accumulation at the thrombus site.^[^ ^14^, ^26^, ^27^, ^28^ ^]^ Traditional rod‐shaped micro motors are usually prepared through complex chemical reactions, which are time‐consuming, low yield, high cost, and may introduce toxic substances when administered in vivo.^[^ ^29^, ^30^ ^]^ In contrast, micro‐motors driven by the intrinsic motion characteristics of biological rod‐shaped cells do not require external fuel or field regulation. For example, swimming‐capable *Escherichia coli* can produce directed movement or mechanical tension.^[^ ^31^ ^]^

*Escherichia coli*, which are abundant in nature and easy to culture, have been studied as drug delivery systems for delivering genes, proteins, and small molecule drugs to treat diseases.^[^ ^32^, ^33^, ^34^, ^35^ ^]^ *Escherichia coli Nissle 1917* (EcN), a non‐pathogenic probiotic, can move autonomously through the oscillation of flagella, making it an excellent new type of biological micro‐motor drug delivery carrier.^[^ ^36^, ^37^ ^]^ Researchers have found that EcN has chemotaxis to inflammatory tissue and certain thrombus aggregation characteristics, making it an excellent carrier for thrombus treatment.^[^ ^38^ ^]^ Surface‐modified probiotics can preserve their rod‐shaped structure and most of their mobility.^[^ ^39^, ^40^, ^41^ ^]^ However, engineering EcN to carry thrombolytic drugs while maintaining its mobility, and achieving active thrombus targeting, deep tissue penetration, and long circulation capabilities in vivo, remains challenging.

**Scheme 1:**
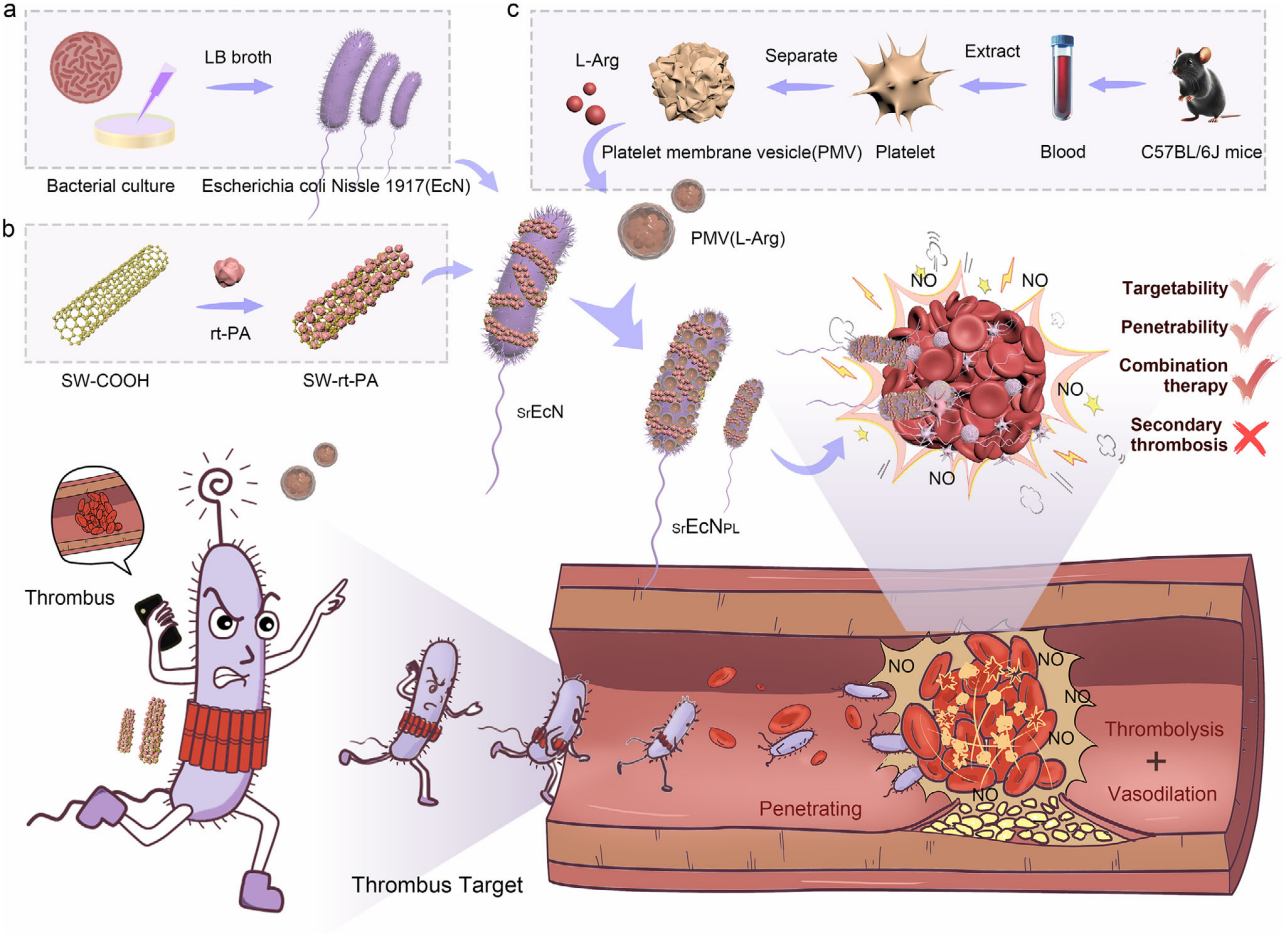
Preparation and thrombolysis mechanism of the engineered probiotic driven micro‐rod robot _Sr_EcN_PL_. a) *E. coli* Nissle 1917 (EcN) is cultivated to obtain a large number of active probiotic colonies. b) The thrombolytic drug rt‐PA is chemically modified onto the surface of SW‐COOH to obtain thrombolytic carbon nanotubes SW‐rt‐PA. c) Platelet cells are extracted from mouse peripheral blood, and platelet membrane vesicle (PMV) are isolated and loaded with L‐Arg to form thrombus targeted drug loaded nanoparticles (PMV(L‐Arg)). The EcN surface is modified with SW‐rt‐PA and PMV(L‐Arg) separately to obtain the engineered probiotic driven micro‐rod robot _Sr_EcN_PL_. The robot targets and deeply penetrates thrombus tissue through platelet membrane and its micro‐rod structure, combining thrombolytic drugs and vasodilators to treat thrombus and inhibit the formation of secondary thrombus.

Herein, this study proposed an engineered probiotic‐powered micro‐rod robot for targeted and penetrative treatment for thrombus. As illustrated in **Scheme** **1**, we developed an engineered probiotic‐driven micro‐rod robot (_Sr_EcN_PL_) through the following fabrication process: 1) utilize natural probiotics as carriers, 2) load thrombolytic single‐walled carbon nanotubes (SW‐rt‐PA), and 3) surface‐functionalizing the probiotics with thrombus‐targeted platelet membrane vesicles encapsulating vasodilators (PMV(L‐Arg)). The _Sr_EcN_PL_ robotic system leverages the low‐immunogenic EcN's exceptional autonomous motility and inflammatory chemotaxis. It integrates platelet membrane vesicles (PMVs) for active thrombus targeting, enables rapid and precise delivery of thrombolytic (rt‐PA) and vasodilators (L‐Arg) payloads to the thrombotic site. Based on the natural rod‐shaped structure of probiotics, it has a smaller size and self‐propulsion ability. This allows for deep penetration into thrombus tissue, enabling the thrombus to decompose from the inside and resulting in faster thrombolysis efficiency. This significantly shortens the thrombolysis time for acute thrombotic diseases and offers new strategies for treating thrombotic diseases with shorter treatment time windows.

Persistent vascular wall injury and thrombolytic drug depletion can lead to secondary thrombus, which is common in atrial fibrillation, acute atherosclerotic cerebral infarction and other diseases. In order to suppress secondary thrombus, this study combines the vasodilator L‐arginine (L‐Arg) with the thrombolytic drug alteplase (rt‐PA). By utilizing the long circulation effect of thrombolysis micro‐robots in vivo and the vasodilation and repair effects of L‐Arg, the blood flow can be maintained for a longer time, thereby inhibiting secondary thrombus formation. In vivo and in vitro results indicated that this micro‐robot can quickly and accurately target and penetrate thrombus tissue. It prolongs the biological half‐life of thrombolytic drugs (≈332 times) and delays secondary thrombus formation. This novel dual‐pronged combined thrombolytic therapy has significant scientific potential for treating vascular injury‐induced thrombotic diseases.

## 2 Results and Discussions

### 2.1 Preparation and Characterization of EcN and SW‐rt‐PA

The preparation methods and mechanisms of engineered probiotic driven micro‐rod robot drug delivery system _Sr_EcN_PL_ as shown in Figure S1 (Supporting Information): 1) First, cultivate EcN and obtain a large number of active probiotic cells. 2) Second, prepare _Sr_EcN: The thrombolytic drug rt‐PA grafted onto the surface of SWCNT‐COOH through acylation to obtain thrombolytic carbon nanotubes SW‐rt‐PA. Then, through acylation reaction, modify SW‐rt‐PA onto the surface of EcN to obtain _Sr_EcN. 3) Finally, prepare _Sr_EcN_PL_: Extract and isolate platelet cells from mouse bone marrow, isolate platelet membranes, and load vasodilator drugs L‐Arg to obtain the thrombotic‐targeted drug loaded nano‐vesicles PMV(L‐Arg). We functionalized _Sr_EcN with PMV(L‐Arg) via streptavidin‐biotin conjugation to obtain micro‐rod robot _Sr_EcN_PL_. This high‐affinity binding strategy ensured high stable surface modification while preserved both the probiotic motility and nanoparticle bioactivity. The micro‐rod robots in this study were fabricated by covalently immobilizing thrombolytic rt‐PA on EcN surfaces. This design enables sustained surface‐localized thrombolytic activity without drug release, allowing probiotics to exert thrombolytic effects while penetrating thrombus tissue, and thus achieving rapid thrombolytic effects.^[^ ^42^, ^43^, ^44^ ^]^

**Figure 1:**
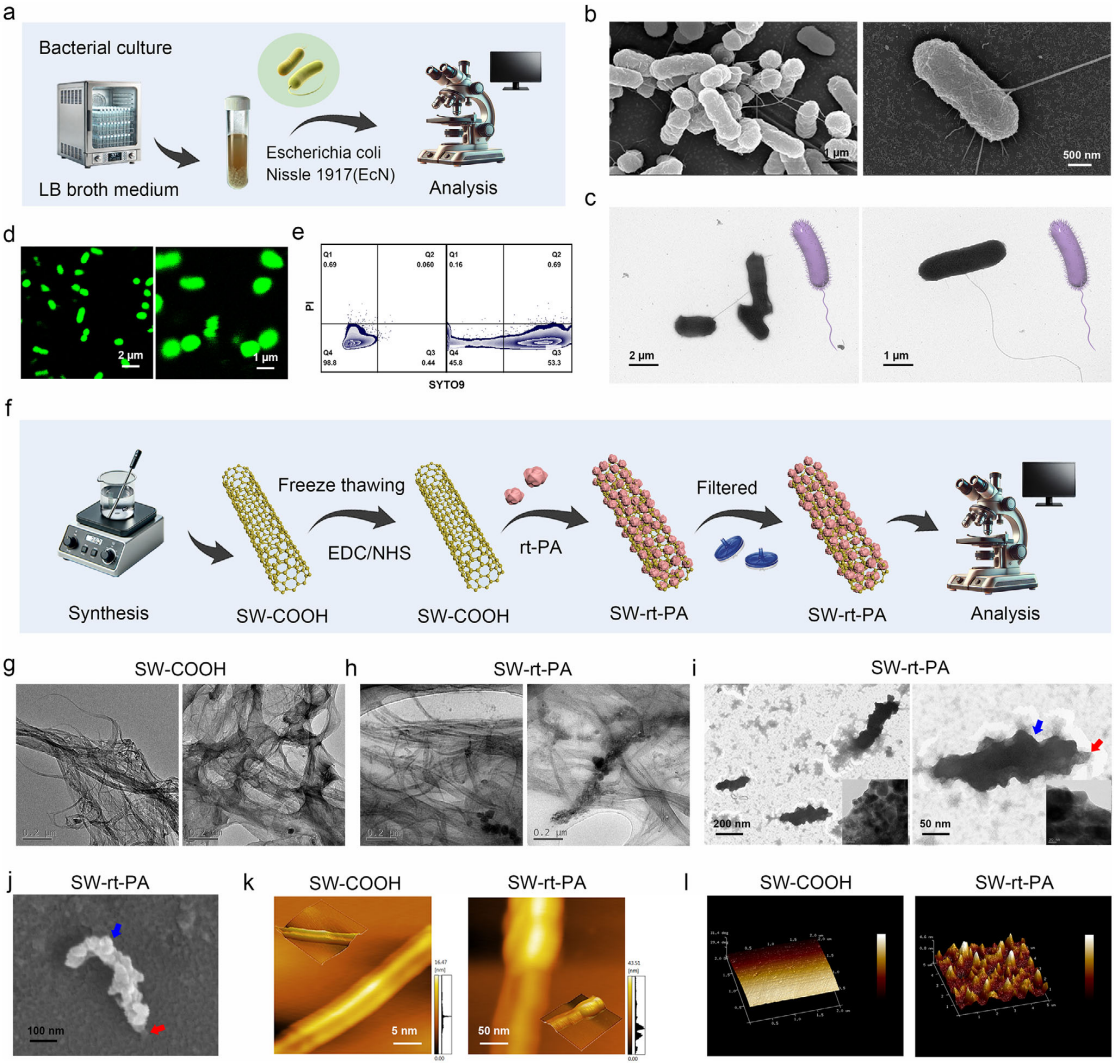
Preparation and characterization of EcN and thrombolytic carbon nanotubes SW‐rt‐PA. a) Non‐pathogenic EcN is cultivated with LB broth medium. b,c) TEM and SEM images of the highly active EcN. d) Analysis of EcN labeled with active probiotic marker dye SYTO9 by laser confocal system. e) Flow cytometric analysis of the survival and mortality rates of EcN. Probe SYTO9 labels live probiotic, while probe PI labels dead probiotic. f) Synthesis route of thrombolytic carbon nanotubes SW‐rt‐PA. g,h) TEM images of SW‐COOH and SW‐rt‐PA (unfiltered). i,j) TEM and SEM images of SW‐rt‐PA after filtered with 0.45 µm filter membrane. The red arrows refer to carbon nanotubes. The blue arrows refer to rt‐PA protein. k,l) Atomic force microscopy (AFM) analysis of the 2D morphology and 3D morphology of SW‐COOH and SW‐rt‐PA.

First, prepare a large quantity of highly active EcN. As shown in the **Figure** **1**a, LB medium used for wet cultivation of EcN. TEM, SEM and laser scanning confocal microscopy (CLSM) images of cultured EcN as shown in Figures 1b,c, and S2 (Supporting Information), indicated that the cultured probiotic showed good morphology, with a rod length of ≈2–3 µm. The live probiotic staining probe SYTO9 was used to stain EcN in Figure 1d, observed under CLSM, and displayed a standard rod‐shaped morphology. To evaluate the survival rate of the cultured EcN, a combination of live and dead probiotic staining probes was used to stain EcN, followed by flow cytometry analysis. As shown in the Figure 1e, the cultured EcN had a high survival rate of ≈93% and a low mortality rate. These results confirm the reliability of the EcN cultivation method utilized in this study.

The preparation steps of thrombolytic single‐walled carbon nanotubes (SWCNT‐rt‐PA = SW‐rt‐PA) are shown in the Figure 1f. First, highly purity carboxylate single‐walled carbon nanotubes ((SWCNT‐COOH = SW‐COOH)) was bought from Nanjing/Jiangsu XFNANO Materials Tech Co., Ltd (101369, diameter 1–2 nm, length 1–3 µm). Then, the SW‐COOH underwent three freeze‐thaw cycles and was activated with EDC/NHS for 1 h. Rt‐PA was added and grafted onto the surface of single‐walled carbon nanotubes through an acylation reaction to obtain SW‐rt‐PA. Finally, a 0.45 µm filter membrane was used to filter SW‐rt‐PA, yielding smaller sized particles. The Raman spectra and IR spectra of SW‐COOH was shown in Figure S3 (Supporting Information), proved the successful synthesis of SW‐COOH. The TEM images of SW‐COOH and SW‐rt‐PA shown in Figure 1g,h, display highly entangled carbon nanotubes, and there are many protein particles on the surface of SW‐COOH. Research has found that such highly aggregation and entangled carbon nanotubes were not conducive to intravenous drug delivery and could cause thrombus in the bloodstream.^[^ ^45^, ^46^, ^47^ ^]^ The biosafety of carbon nanotubes as drug delivery systems can be improved by changing the size, aggregation, and surface modification of carbon nanotubes.^[^ ^48^, ^49^, ^50^, ^51^ ^]^ Through repeated freeze‐thaw cycles method to crush SW‐COOH. By filtering SW‐rt‐PA, obtained a smaller particle size and lower aggregation drug loading single‐walled carbon nanotubes, improved biological safety of SW‐rt‐PA is beneficial for blood administration. TEM and SEM images of SW‐rt‐PA as shown in the Figure 1i,j, revealed that extensive protein wrapped around the surface of carbon nanotubes. The thrombolytic carbon nanotube SW‐rt‐PA had a length of ≈200–300 nm and a width of ≈50–100 nm. The AFM images of SW‐rt‐PA as shown in the Figure 1k,l, showed that the surface of SW‐COOH was smooth and narrow, while the surface of SW‐rt‐PA had obvious protein modification, resulting in a significant increase in width. The encapsulation efficiency and drug loading capacity of rt‐PA onto SW‐COOH nanotubes were quantitatively analyzed using protein quantification methods. The drug entrapment efficiency of rt‐PA on SW‐COOH was 89.21%, and the drug loading capacity was 35.23%. The above results demonstrate the successful preparation of thrombolytic carbon nanotubes SW‐rt‐PA.

### 2.2 Preparation and Characterization of PMV(L‐Arg)

**Figure 2:**
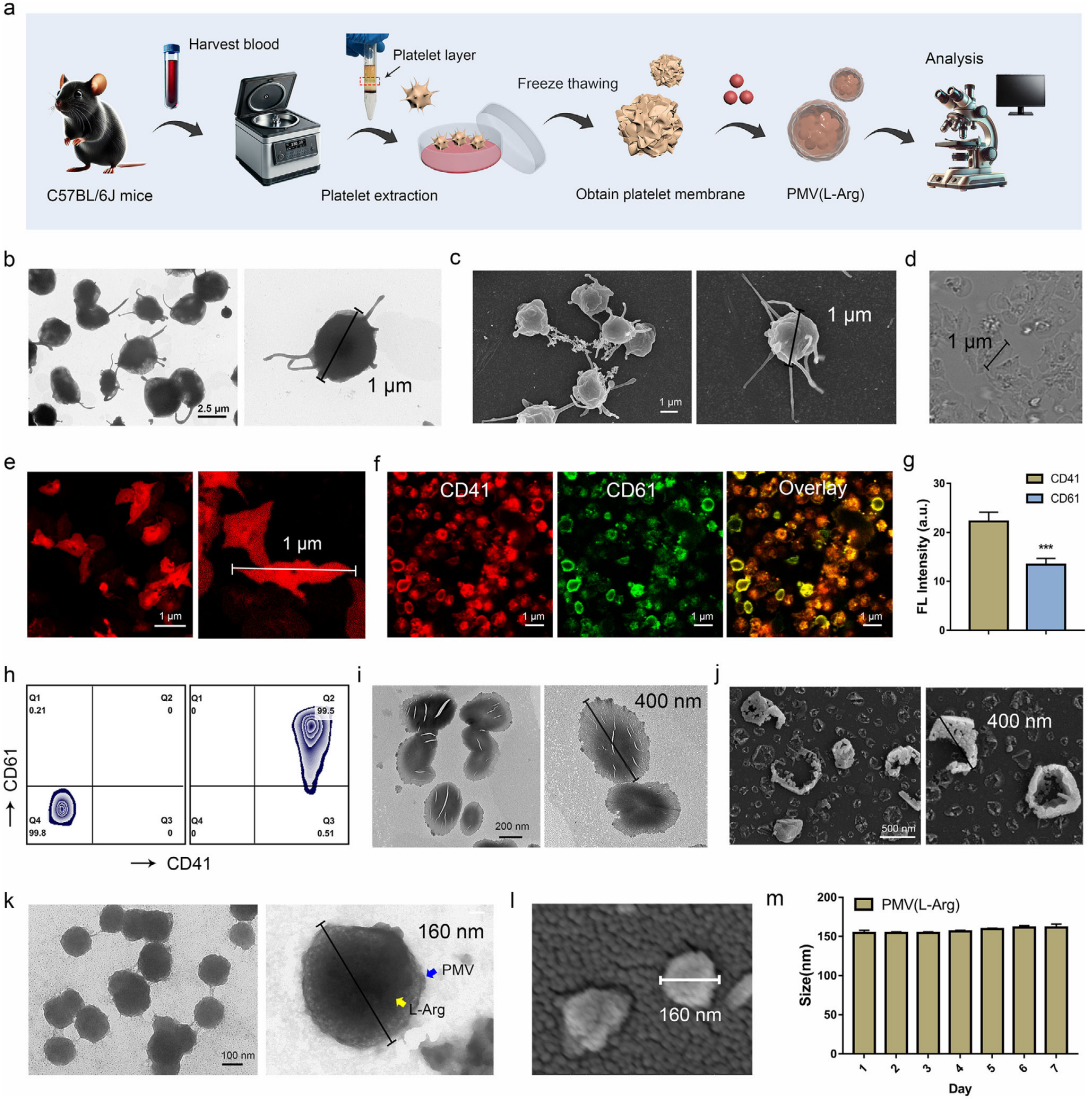
Preparation and characterization of thrombus targeted drug loaded nanoparticles (PMV(L‐Arg)). a) The preparation route of drug loaded nanoparticles PMV(L‐Arg). b,c) TEM and SEM images of platelet extracted from mouse peripheral blood. d) Analysis of platelets with laser scanning confocal microscopy system. e) Fluorescently labeled platelets with probe DiR analyzed by a laser scanning confocal microscopy system. f,g) Immunofluorescence analysis of the characteristic proteins (CD61 and CD41) express on the platelet surface. (n = 3; mean ± SD). ***P < 0.001. h) Flow cytometric analysis of the purity of platelets stained with CD61 and CD41 antibodies. i,j) TEM and SEM images of platelet membranes extracted from platelets. k) TEM images of platelet membrane drug loaded nanoparticles PMV(L‐Arg). l) SEM and dynamic light scattering data (DLS) images of platelet membrane drug loaded nanoparticles (PMV(L‐Arg)). (m) Particle size stability of PMV(L‐Arg) nanoparticles in 10 mM PBS for one week at 4 °C. (n = 3; mean ± SD).

Platelets possess thrombotic aggregation properties, and drug loaded nanoparticles coated with platelet membranes exhibit thrombotic tissue targeting properties. As shown in **Figure** **2**a: First, platelet cells were isolated and purified from mouse peripheral blood; and the platelet membranes were obtained through three freeze‐thaw cycles; Finally, vasodilator L‐Arg was loaded into the platelet membranes to produce the thrombus‐targeted drug‐loaded nanoparticles PMV(L‐Arg). TEM and SEM images of platelet extracted from mouse peripheral blood as shown in Figures 2b,c and S4 (Supporting Information), showed that mostly platelet cells exhibited an oval shaped appearance with multiple pseudopodia. The cell membrane fluorescent probe DiR used to stain the platelet membrane, as shown in the Figures 2d and S4 (Supporting Information), revealing the typical nuclear free structure of platelets. To verify that the extracted cells are platelets, flow cytometry was used to analyze the expression of platelet characteristic proteins CD61 and CD41. As shown in Figure 2e, ≈99.5% of cells co‐expressed CD61 and CD41. Immunofluorescence (Figure 2f; Figure S5, Supporting Information) and fluorescence co‐localization studies (Figure S6, Supporting Information) demonstrated co‐expression of CD61 and CD41 in the majority of cells, confirming successful isolation of platelet populations with well‐preserved morphological characteristics.

The platelet membranes were extracted using three consecutive repeated freeze‐thaw cycles with liquid nitrogen and a 37 °C‐water bath. TEM and SEM images of the extracted platelet membranes as shown in Figure 2h,i, demonstrated that this method obtained platelet membranes with excellent film‐forming properties. An ultrasonic disruptor was used to treat platelet membranes and load vasodilator L‐Arg, resulting in the thrombus‐targeted drug‐loaded nanoparticles PMV(L‐Arg). As shown in Figure 2j,k, the PMV(L‐Arg) nanoparticles exhibited a distinct core‐shell structure, with a spherical shape and uniform particle size of ≈150 nm. The particle size and zeta potential of PMV(L‐Arg) nanoparticle detected by dynamic light scattering data (DLS) as shown in Figure S7 (Supporting Information), indicating that the particle size was ≈156.21 ± 2.5 nm, zeta potential was ≈ −6.3 ± 1.1 mv. The drug entrapment efficiency of L‐Arg from platelet membrane was 88.34%, and the drug loading capacity was 38.62%. The particle size stability of PMV(L‐Arg) nanoparticles was evaluated by DLS as Figure 2l, indicating that the nanoparticles were relatively stable. The above results demonstrate the successful preparation of thrombus‐targeted drug‐loaded nanoparticles PMV(L‐Arg).

### 2.3 Preparation and Characterization of _Sr_EcN_PL_

**Figure 3:**
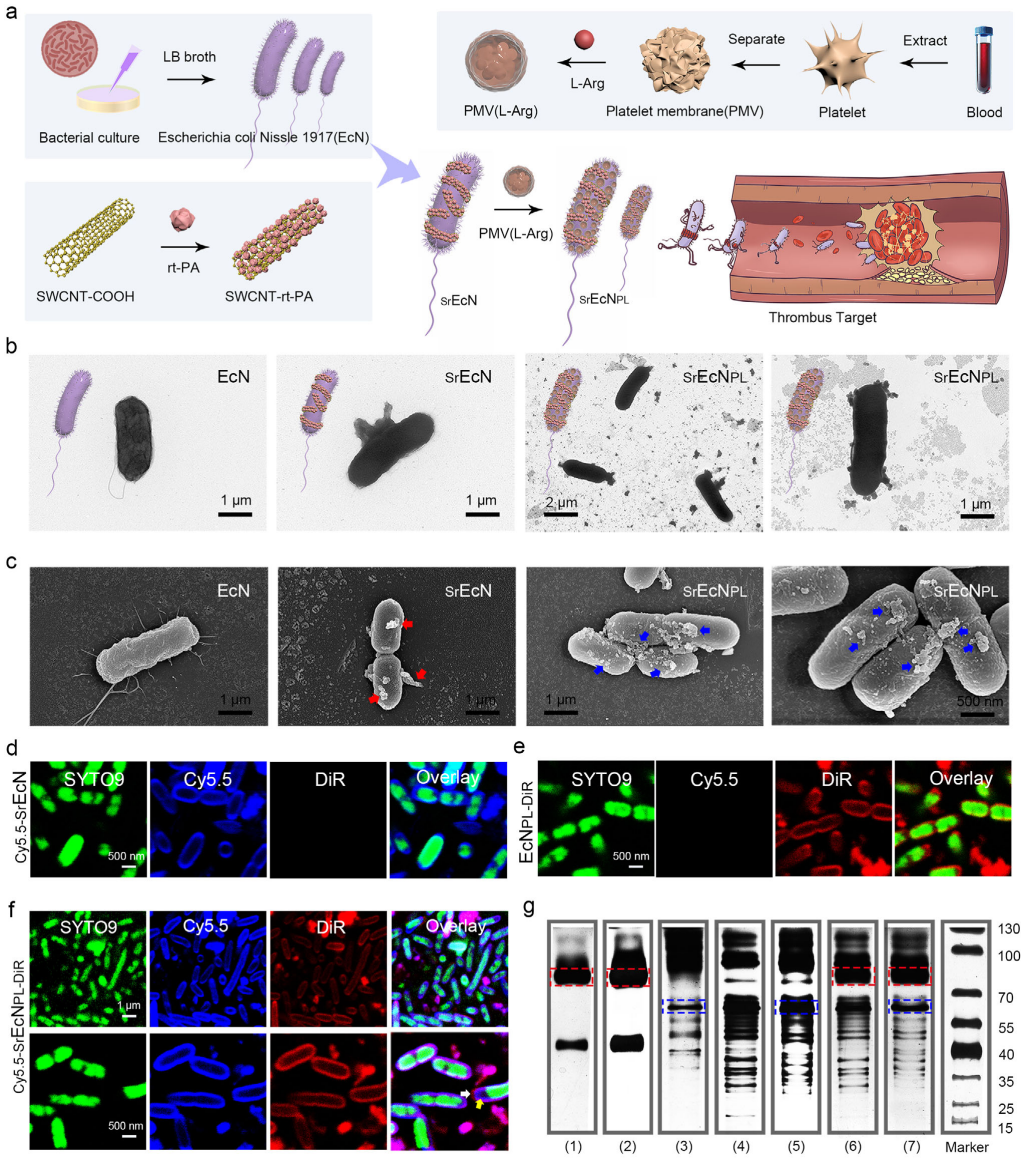
Preparation and characterization of the engineered probiotic driven micro‐rod robot _Sr_EcN_PL_. a) The preparation route of the engineered probiotic driven micro‐rod robot _Sr_EcN_PL_. b,c) TEM and SEM images of EcN, SW‐rt‐PA modified EcN (_Sr_EcN) and PMV(L‐Arg) modified EcN (_Sr_EcN_PL_). The red arrows refer to SW‐rt‐PA. The blue arrows refer to SW‐rt‐PA and PMV(L‐Arg). d–f) A laser confocal fluorescence imaging system was used to analyze different micro‐rod (EcN, _Sr_EcN and _Sr_EcN_PL_). Green fluorescence refers to probiotic labeled with probe SYTO9. Blue fluorescence refers to SW‐rt‐PA labeled with probe Cy5.5. Red fluorescence refers to PMV(L‐Arg) labeled with probe DiR. The white arrows refer to SW‐rt‐PA. The yellow arrows refer to PMV(L‐Arg). g) SDS‐PAGE was used to separate and detect the proteins in different components (1): rt‐PA, 2): SW‐rt‐PA, 3): PMV(L‐Arg), 4): EcN, 5): EcN_PL_, 6): _Sr_EcN, 7): _Sr_EcN_PL_, Marker). The red boxes refer to the characteristic protein bands of rt‐PA. The blue boxes refer to the characteristic protein bands of PMV(L‐Arg).

The preparation of micro‐rod robot _Sr_EcN_PL_ as shown in the **Figure** **3**a and was conducted following the methods outlined in Figure S1 (Supporting Information). TEM and SEM images of micro‐rod robot _Sr_EcN_PL_ as shown in the Figure 3b,c, showed that there were some tubular proteins like SW‐rt‐PA modified on the surface of EcN in images of _Sr_EcN group; and there were many organic particles like PMV(L‐Arg) modified on the surface of EcN in images of _Sr_EcN_PL_ group. To demonstrate the successful surface modification of EcN with SW‐rt‐PA and PMV(L‐Arg), the prepared _Sr_EcN_PL_ was observed by fluorescence imaging method. Rt‐PA proteins were labelled with to produce SW‐rt‐PA‐Cy5.5, and the cell membrane staining probe DiR was used to stain platelet membranes to prepare DiR‐PMV(L‐Arg). These labeled components were separately modified onto EcN to obtain _Cy5.5‐Sr_EcN_,_ EcN_PL‐DiR,_ and _Cy5.5‐Sr_EcN_PL‐DiR_, with EcN stained using the live probiotic staining probe SYTO9. As shown in the Figure 3d, Figure S8 (Supporting Information), the surface of the green, fluorescent probiotic cells had abundant blue fluorescence, indicating that the SW‐rt‐PA successfully modified on the surface of EcN. Similarly, Figure 3e, Figure S8 (Supporting Information) displayed extensive red fluorescence on the surface of the green, fluorescent probiotic cells, confirming the PMV(L‐Arg) successfully modified on the surface of EcN. Figure 3f further demonstrated that both blue and red fluorescence are present on the surface of the green probiotic cells, with blue fluorescence enveloping the green fluorescence and red fluorescence enveloping the blue fluorescence, proving the successful and distinct modification of SW‐rt‐PA and PMV(L‐Arg) onto EcN.

The drug entrapment efficiency (EE) and drug loading capacity (DL) of SW‐rt‐PA and PMV(L‐Arg) in _Sr_EcN_PL_ were assessed using fluorescence analysis. SW‐rt‐PA‐Cy5.5 and DiR‐PMV(L‐Arg) was prepared to obtain _Cy5.5‐Sr_EcN_PL‐DiR_, and EcN was stained with the probe SYTO9. The results shown in the Figure S9 (Supporting Information), the EE of SW‐rt‐PA and PMV(L‐Arg) in _Sr_EcN_PL_ is relatively high (EE(SW‐rt‐PA): 90.73%, EE(PMV(L‐Arg)): 82.17%). The corresponding DL of SW‐rt‐PA and PMV(L‐Arg) in _Sr_EcN_PL_ were DL(SW‐rt‐PA):38.53% and DL(PMV(L‐Arg)):17.74%. Based on the drug loading of rt‐PA and L‐Arg in SW‐rt‐PA and PMV(L‐Arg), the drug loading of rt‐PA and L‐Arg in _Sr_EcN_PL_ are 13.57% and 6.85%, the ratio of rt‐PA compared with L‐Arg was ≈2:1. This ratio is rational given that rt‐PA, the main thrombolytic drug, requires a larger amount, while L‐Arg serves as the adjuvant drug used to dilate blood vessels. These results indicated that the methods used in this study to modify SW‐rt‐PA and PMV(L‐Arg) on the surface of EcN is reasonable and successful.

The retention activity of rt‐PA in SW‐rt‐PA and _Sr_EcN_PL_ was compared with the activity of corresponding concentration of free rt‐PA using the chromogenic subtract S‐2288. As shown in the Figure S10 (Supporting Information), the retention activity of rt‐PA in SW‐rt‐PA is 78%, and the retention activity of rt‐PA in _Sr_EcN_PL_ is 66%, which may be due to the damage caused by the process of modifying probiotics with SW‐rt‐PA and PMV(L‐Arg). Sodium dodecyl sulfate‐Polyacrylamide gel electrophoresis (SDS‐PAGE) was used to separate and detect the proteins in different components (((1): rt‐PA, (2): SW‐rt‐PA, (3): PMV(L‐Arg), (4): EcN, (5): EcN_PL_, (6): _Sr_EcN, (7): _Sr_EcN_PL_, Marker).) shown in Figure 3g, the result also proved that SW‐rt‐PA and PMV(L‐Arg) had been successfully modified on the surface of EcN. The above results fully demonstrate the successful preparation of _Sr_EcN_PL_, proved that the synthetic experimental method used is correct.

### 2.4 Functional Characterization of _Sr_EcN_PL_

**Figure 4:**
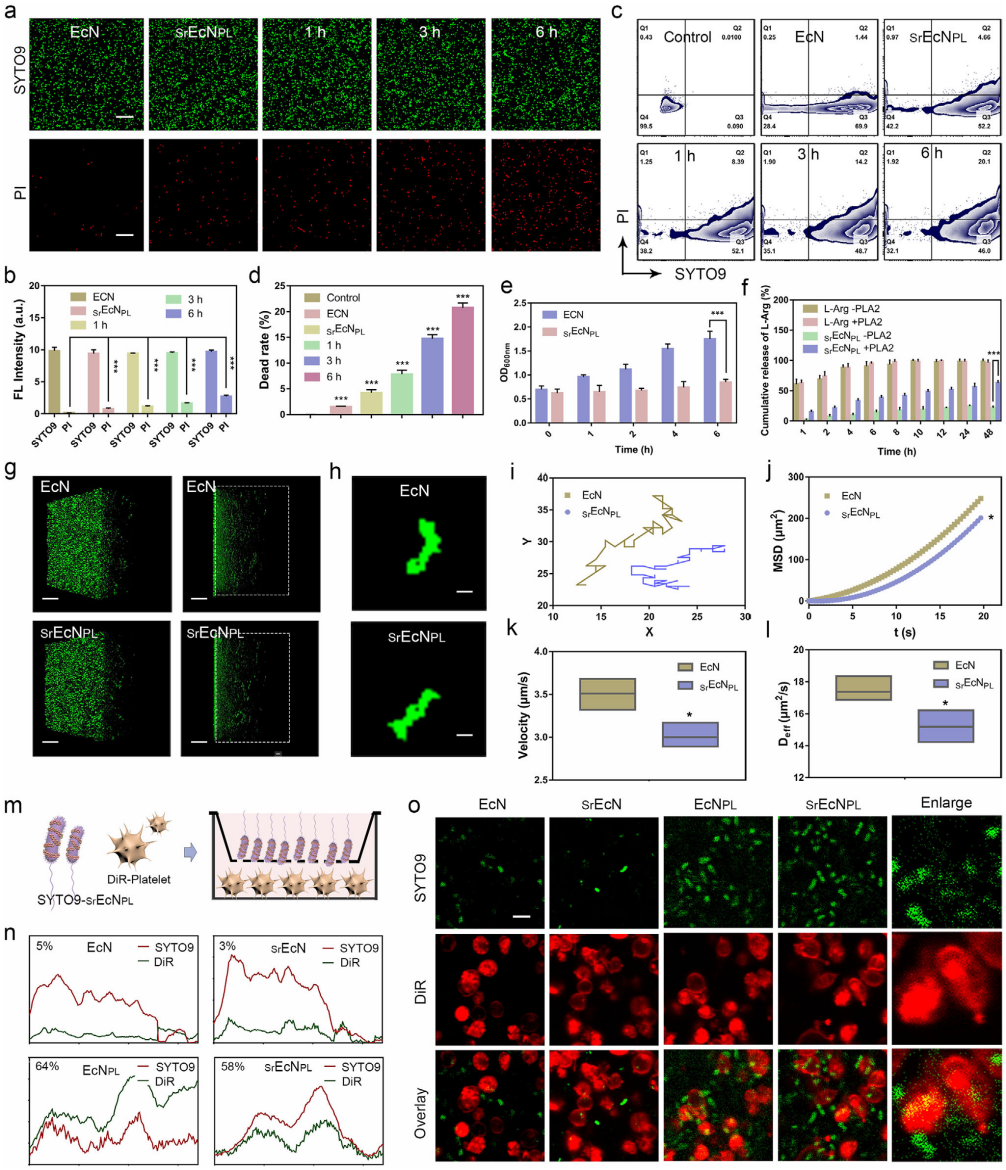
Function characterization of the engineered probiotic driven micro‐rod robot _Sr_EcN_PL_. a,b) A laser confocal fluorescence imaging system was used to analyze the survival and dead rates of EcN and _Sr_EcN_PL_ in LB broth medium. Probe SYTO9 labels live probiotic, and probe PI labels dead probiotic. Scale bars, 100 µm. (n = 3; mean ± SD). ***P < 0.001. c,d) Flow cytometric analysis of the survival and dead rates of EcN and _Sr_EcN_PL_ in LB broth medium at different time points. Probe SYTO9 labels live probiotic, and probe PI labels dead probiotic. (n = 3; mean ± SD). ***P < 0.001. e) The OD_600_ _nm_ values of different probiotics (EcN, _Sr_EcN_PL_) at different time points (0, 1, 2, 3, 4, 5, and 6 h). (n = 3; mean ± SD). ***P < 0.001. f) Cumulative release of L‐Arg from L‐Arg and _Sr_EcN_PL_ robot in the medium with or without PLA2. (n = 3; mean ± SD). ***P < 0.001. g) The 3D fluorescence images of EcN and _Sr_EcN_PL_ labeled with probe SYTO9, analyzed by confocal z‐axle layer scanning mode. h) The fluorescence superposition images of motion trajectories of EcN and _Sr_EcN_PL_ labeled with probe SYTO9 over 20 s. Scale bars, 10 µm. i) Motion behavior of EcN and _Sr_EcN_PL_ in 10% FBS medium: Optical tracking trajectories (20 s). j) MSD of EcN and _Sr_EcN_PL_ in 10% FBS medium. (n = 3; mean ± SD). *P < 0.05. k) The average migration speed of EcN and _Sr_EcN_PL_ in 10% FBS medium. (n = 3; mean ± SD). *P < 0.05. l) Deff of EcN and _Sr_EcN_PL_ in 10% FBS medium (n = 3). (n = 3; mean ± SD). *P < 0.05. m) Schematic diagram of in vitro platelet targeting experiment of _Sr_EcN_PL_ robot. n,o) Fluorescence imaging and fluorescence co‐localization analysis of different probiotics (EcN, _Sr_EcN, EcN_PL_, _Sr_EcN_PL_) targeting platelets for 3 h. Probe SYTO9 labels live probiotics, and probe DiR labels platelets. Scale bars, 2 µm.

To investigate the probiotic survival rate of prepared micro‐rod robot _Sr_EcN_PL_, the live/dead bacteria double stain kit (Shanghai Maokang Biotechnology Co., Ltd., Shanghai, China, MX 4234) was used to analysis. As shown in the **Figure** **4**a,b, confocal fluorescence imaging system was used to analyze the survival rate of _Sr_EcN_PL_. The red fluorescence intensity of _Sr_EcN_PL_ was higher than that of EcN, indicating that the modifying SW‐rt‐PA and PMV(L‐Arg) on the surface of EcN will reduced the survival rate of EcN. Over time, the intensity of red fluorescence gradually increased, suggesting a gradual rise in probiotic mortality (Figure S11, Supporting Information). Flow cytometry was used to analyze the dead rate of _Sr_EcN_PL_. As shown in the Figure 4c,d, the dead rate of _Sr_EcN_PL_ (4.66%) was higher than that of EcN (1.44%), likely due to the modification operation of SW‐rt‐PA and PMV(L‐Arg), which may have compromised bacterial viability. The death rate of _Sr_EcN_PL_ gradually increased over time, reached 20% at 6 h. The OD_600_ _nm_ measurement of EcN and _Sr_EcN_PL_ (Figure 4e) showed that the concentration of probiotic solution _Sr_EcN_PL_ was significantly lower than that of EcN over time. The above results indicate that the dead rate of _Sr_EcN_PL_ modified with SW‐rt‐PA and PMV(L‐Arg) increases, and its reproductive ability is significantly inhibited. This may be due to the large number of tubular modifications wrap around the probiotic membrane, which makes it difficult for probiotic to divide and reproduce normally.

Apolipoprotein‐associated phospholipase A2 (PLA2), also known as platelet activating factor acetylhydrolase (PAF‐AH), can hydrolyze platelet membranes.^[^ ^52^, ^53^ ^]^ PLA2 is an independent risk factor for endothelial inflammation and a new indicator for detecting endothelial inflammation, which highly expressed at the site of thrombus.^[^ ^54^, ^55^ ^]^ Therefore, PLA2 level used to evaluate the drug release ability of _Sr_EcN_PL_ at thrombus site. As shown in the Figure 4f, under the condition of the presence of PLA2 (200 ng mL^−1^), the release dose of _Sr_EcN_PL_ significantly increased compared to without PLA2 condition as times goes on. About 70% of the L‐Arg drugs released within 48 h, indicating that the drug release of _Sr_EcN_PL_ was clearly responsive to PLA2. According to the synthesis method of _Sr_EcN_PL_, SW‐rt‐PA and EcN were covalently bound, which was a stable binding mode. Therefore, rt‐PA remained attached to _Sr_EcN_PL_, enabling probiotics to exert thrombolytic effects while penetrating thrombus tissue for rapid thrombolysis.^[^ ^42^, ^56^, ^57^ ^]^ To investigate the exposure content of rt‐PA from _Sr_EcN_PL_, a human rt‐PA ELISA kit was used to analyze the content changes of rt‐PA exposed on the surface of _Sr_EcN_PL_ robot under different media conditions (with or without PLA2, 200 ng mL^−1^). As shown in the Figure S12 (Supporting Information), there was no significant change in the content of rt‐PA in the absence of PLA2. However, the content of rt‐PA increased significantly in the presence of PLA2 within 48 h, which might be due to the shielding of some rt‐PA (≈25%) by platelet membrane drug loaded nano‐vesicles PMV(L‐Arg) on the surface of EcN. This indicated that the exposure of rt‐PA form _Sr_EcN_PL_ robot also exhibited partial thrombus tissue‐specific responsive ability. The above results demonstrate that the _Sr_EcN_PL_ system has thrombotic tissue responsive drug release properties, provided a basis for the L‐Arg drug to exert vasodilation and inflammatory repair effects.

This study constructs a micro‐rod‐shaped robot _Sr_EcN_PL_ for targeted treatment of thrombus by modifying natural rod‐shaped probiotics. Rod shaped probiotics with flagella motors exhibit rapid self‐driven movement. In order to investigate the changes in the motility of EcN modified with SW‐rt‐PA and PMV(L‐Arg), a 100× objective magnification laser confocal microscope was used to observe and record the motion behaviors of _Sr_EcN_PL_ robot. The 3D fluorescence images of EcN and _Sr_EcN_PL_ labeled with probe SYTO9 were analyzed by confocal z‐axle layer scanning mode as shown in the Figure 4g and Movie S1 (Supporting Information), indicating that within the same scanning plane, the motion ranges of EcN and _Sr_EcN_PL_ were similar. The motion trajectory and corresponding coordinate of EcN and _Sr_EcN_PL_ in Figure 4h and Movie S2 (Supporting Information) were analyzed by Fiji software shown in Figure 4i. The length of the motion trajectory of _Sr_EcN_PL_ was shorter than EcN within the same period. The mean‐squared displacement (MSD), effective diffusion coefficient (Deff) and average migration velocity of particles were analysed by MATLAB software refer to previous studies.^[^ ^58^, ^59^ ^]^ As shown in Figure 4j–l, the MSD, velocity and Deff of _Sr_EcN_PL_ were all smaller than EcN, which might due to the modification of EcN by SW‐rt‐PA and PMV(L‐Arg) disrupting some flagella structures on the surface of EcN and reducing its mobility. However, _Sr_EcN_PL_ still retained most of its motility, and its micro‐rod structure was conducive to penetrate thrombus.

To investigate whether dead probiotics have the ability to motion and still have the potential for drug delivery, we studied the motion ability of dead probiotics. As shown in the Figure S13 and Movie S3 (Supporting Information), the dead probiotics (labeled with probe PI), the morphology of the dead probiotics exhibited structural distortion, cell wall damage, content outflow, and easy aggregation. The motion trajectory and MSD values showed that the dead probiotics exhibited diffusive Brownian motion characteristics and lacked autonomous movement ability. Therefore, dead probiotics lack the potential for active thrombolytic drug delivery for rapid targeted thrombolysis.

To investigate the in vitro targeting ability of _Sr_EcN_PL_, transwell chambers were used. The experimental diagram was shown in Figure 4m. Purified platelet cells were cultured at the bottom of the culture dishes, and different groups were added to the upper chamber with a pore size of 0.45 µm and incubate for 3 h. The live EcN was labeled with the probe SYTO9, and platelets with the probe DiR. These results (Figure 4n,o; Figure S14, Supporting Information) showed that the quantity of probiotics reached the lower chamber were different in four groups. EcN and _Sr_EcN had similar platelet‐targeting abilities, both significantly lower than that of EcN_PL_, indicating that PMV(L‐Arg) provided platelet‐targeting ability to EcN. In addition, the platelets target ability of EcN_PL_ was slightly higher than _Sr_EcN_PL_, which might due to the more complex surface modifications, the movement and targeting ability of probiotics were slightly inhibited. The above results demonstrate that _Sr_EcN_PL_ robot has significant platelet targeting ability, establishing the foundation for the in vivo thrombus targeting movement of robot.

### 2.5 In Vitro Thrombolysis Efficacy and In Vivo Thrombus Targeting Evaluation of _Sr_EcN_PL_

**Figure 5:**
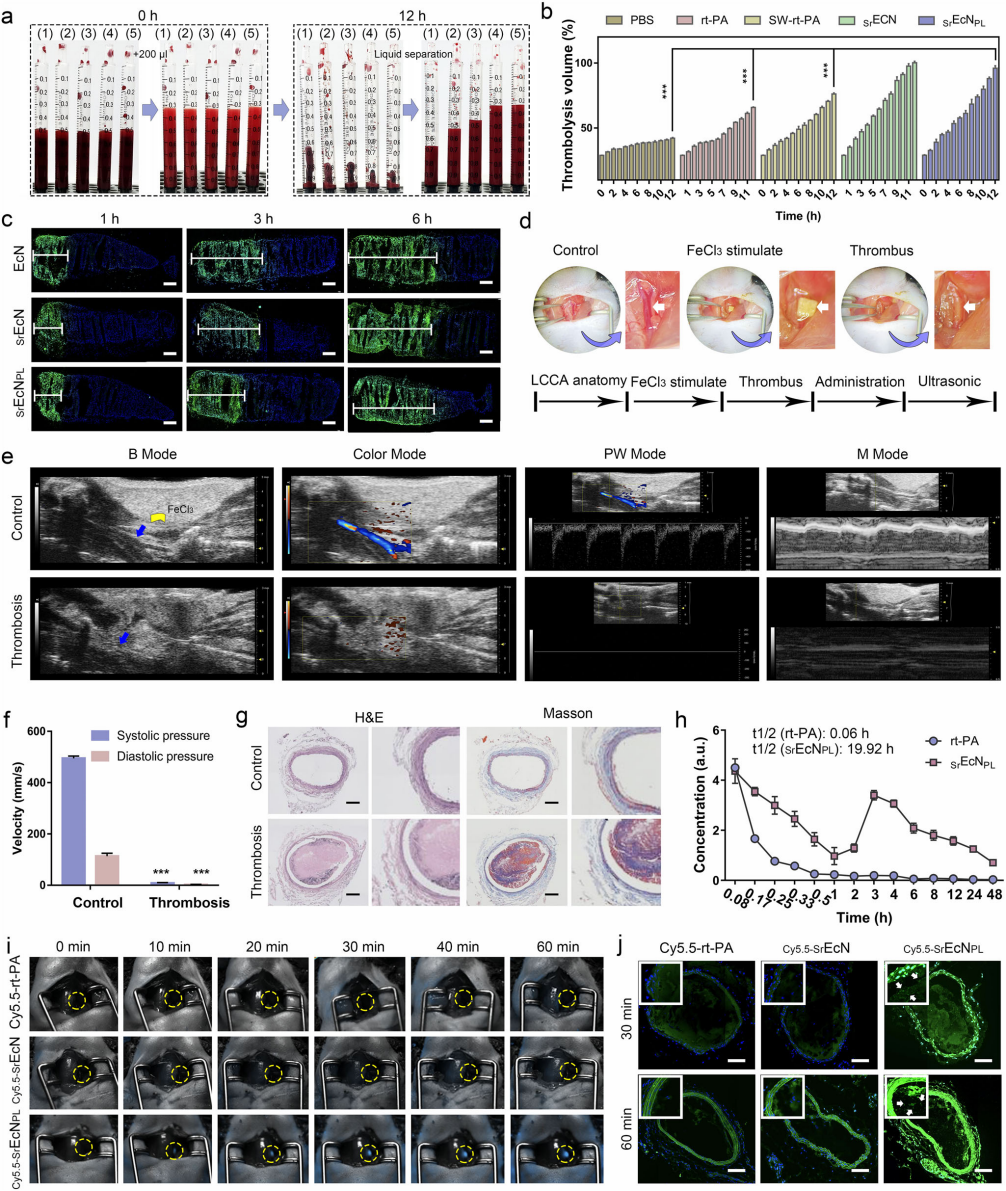
In vitro thrombolysis and in vivo targeting evaluation of _Sr_EcN_PL_ robot. a,b) Thrombolysis efficacy evaluation of different groups (1): PBS, 2): rt‐PA, 3): SW‐rt‐PA, 4): _Sr_EcN, 5): _Sr_EcN_PL_) for 12 h in vitro. (n = 3; mean ± SD). ***P < 0.001. c) The fluorescence microscopy analysis of the penetration ability of different probiotic (EcN, _Sr_EcN, _Sr_EcN_PL_) into blood clots at various time points. Scale bars, 100 µm. d) Construction of left common carotid artery (LCCA) thrombus model and the experimental scheme of the thrombolysis efficacy evaluation of _Sr_EcN_PL_ robot. e) The left common carotid artery thrombus model was constructed and analyzed by multiple ultrasound imaging modalities. The blue arrows refer to the FeCl_3_ patch location. f) Changes in systolic and diastolic blood velocity of LCCA before and after thrombus model construction. (n = 3; mean ± SD). ***P < 0.001. g) H&E and Masson staining analysis of blood vessels in thrombus model and control group. Scale bars, 100 µm. h) Pharmacokinetic study of rt‐PA and _Sr_EcN_PL_ robot over 48 h (n = 5; mean ± SD). i) Targeting analysis of the left common carotid artery vessels for different groups (Cy5.5‐rt‐PA, _Cy5.5‐Sr_EcN, _Cy5.5‐Sr_EcN_PL_) at different time points. j) Fluorescence imaging analysis of LCCA vessels from different groups (Cy5.5‐rt‐PA, _Cy5.5‐Sr_EcN, _Cy5.5‐Sr_EcN_PL_) at different time points. Scale bars, 100 µm.

In order to investigate the in vitro thrombolysis efficacy of _Sr_EcN_PL_ robot, equal volumes of fresh blood were placed into a syringe tube for evaluating the thrombolytic effect in different groups. As shown in **Figure** **5**a, after coagulation, different groups of samples were added: 1) PBS, 2) rt‐PA, 3) SW‐rt‐PA, 4) _Sr_EcN, and 5) _Sr_EcN_PL_, all at 1.5 × 10^4^ CFU mL^−1^, equivalent to 0.48 mg mL^−1^ rt‐PA and 0.23 mg mL^−1^ L‐Arg. After 12 h incubation at room temperature, solid‐liquid separation was performed once per hour, and the volume of liquid blood obtained from thrombolysis was recorded. The thrombolysis results shown in Figure 5b, the thrombolysis volume in each group showed an increase trend over time. At 12 h, the thrombolysis volume of SW‐rt‐PA and rt‐PA was significantly higher than control PBS. And SW‐rt‐PA was slightly higher than rt‐PA, which might due to the rod‐shaped structure of SW‐rt‐PA, it is prone to rotation and sliding within the thrombus tissue, increase the mass transport of thrombolytic drugs rt‐PA, thereby increasing their utilization and thrombolytic efficiency.^[^ ^60^, ^61^, ^62^ ^]^ The thrombolysis volumes of _Sr_EcN and _Sr_EcN_PL_ were similar, and significantly higher than that of SW‐rt‐PA and rt‐PA. This could be attributed to the self‐propelling ability of EcN and its small size with natural rod‐shaped structure, which facilitated penetration into thrombus tissue, thereby enhanced the thrombolytic effect of rt‐PA from _Sr_EcN_PL_. The hemolytic abilities of different samples in vitro were tested as shown in Figure S15 (Supporting Information), the hemolysis rates of all the samples except water were below 5%, indicating the good blood compatibility of the samples.

To evaluate the thrombus penetration ability of _Sr_EcN_PL_ robot, EcN stained with the probe SYTO9, and thrombus blocks from the syringe at different time points were removed, frozen and sliced, and observed under a confocal microscope. As shown in Figure 5c, Figure S16 (Supporting Information), the depth of penetration of three groups of probiotics into the thrombus block increases with time, the penetration depth of _Sr_EcN_PL_ was lower than that of EcN and EcN_PL_, while the penetration depth of EcN was the highest. This might be due to the complex surface modifications of probiotics interfering with some of their physiological movements, and the thrombus penetration effect of _Sr_EcN_PL_ robot primarily relied on the motility of EcN. The above results demonstrate the excellent thrombus penetration and thrombolytic effect of the _Sr_EcN_PL_ robot. This mechanism is based on the autonomous movement ability of probiotics combined with surface functionalization with thrombolytic agents, which contributes to the thrombolytic effect of _Sr_EcN_PL_ during self‐propelled motion. Clot dissolution facilitates deeper penetration of _Sr_EcN_PL_ robots into thrombi, enhancing localized therapeutic effects. The effective thrombus penetration and thrombolytic activity of the _Sr_EcN_PL_ demonstrate the feasibility of this probiotic‐based drug delivery strategy for thrombolytic therapy.

To evaluate the thrombus targeting and thrombolytic effect of _Sr_EcN_PL_ robot in vivo, an in vivo thrombus model was established using the method shown in the Figure 5d. First, the left common carotid artery blood vessel of C57BL/6 mice was surgical exposures. Then, 10% FeCl_3_‐soaked filter paper was attached to the bifurcation of the left common carotid artery (LCCA) blood vessels for 5 min.^[^ ^22^ ^]^ After removing the filter paper for 10 min, emboli could be clearly observed at the bifurcation of the blood vessels. The wound was sutured, and pre‐ and post‐administration vascular conditions were analyzed via ultrasound imaging. Small‐animal ultrasound imaging was employed to analyze the left common carotid artery (LCCA) before and after thrombus model establishment, utilizing multiple analytical models. As shown in Figure 5e, Movies S4 and S5 (Supporting Information), it could be clearly observed that the blood vessels at the bifurcation of the carotid artery become narrowed after the construction of the thrombus model in B mode, which was also confirmed by M mode. Colour Doppler mode and pulse‐wave (PW) mode further indicated that the blood flow at the bifurcation of the LCCA blood vessels significantly decreased and tended to disappear before and after the construction of the thrombus model (Figure 5f). H&E and Masson staining analysis was performed on the LCCA blood vessels of the thrombus model and the sham blood vessels. As shown in the Figure 5g, the results confirmed successful thrombus model construction, with vessels nearly filled with emboli.

To verify that the _Sr_EcN_PL_ robot drug delivery system constructed in this study serves as an effective drug carrier, its ability to significant prolong the circulation time of rt‐PA (which was covalently modified on the robot's surface) was assessed. This modification helps avoid the increased toxicity and side effects associated with repeated administrations, while the sustained thrombolytic effect further suppresses secondary thrombus formation. The pharmacokinetic experiments were used to analyze the biological half‐life of rt‐PA and _Sr_EcN_PL_ as shown in Figure 5h. The drug concentration‐time curve showed a second peak, which might be due to the rupture of nano vesicles PMV(L‐Arg) on the surface of EcN (the shield of some rt‐PA by PMV(L‐Arg) on the surface of EcN), leading to an increase in drug concentration of rt‐PA in the blood. The biological half‐life of rt‐PA was particularly short ≈0.06 h, while the biological half‐life of _Sr_EcN_PL_ was significantly prolonged up to 19.92 h. The mean retention time of the _Sr_EcN_PL_ was 62.85, which was almost 63 times that of free rt‐PA (Table S1, Supporting Information). The above results demonstrate that _Sr_EcN_PL_ robot has a long circulation effect in the body, which can significantly increase the biocompatibility of rt‐PA, achieve sustained thrombolytic effect, and inhibit the formation of secondary thrombus.

To investigate whether _Sr_EcN_PL_ robot can target thrombus tissue in vivo, a LCCA thrombus model was constructed. Thrombotic model mice were administered different samples (Cy5.5‐rt‐PA, _Cy5.5‐Sr_EcN, _Cy5.5‐Sr_EcN_PL_, 1.5 × 10^4^ CFU mL^−1^, equal to 0.48 mg mL^−1^ rt‐PA) through the tail vein and observed by a small animal imaging device for 1 h. The results as shown in Figure 5i, Figure S17 (Supporting Information), there almost no fluorescence was observed at the thrombus site in groups Cy5.5‐rt‐PA and _Cy5.5‐Sr_EcN of mice within 1 h. However, significant fluorescence observed at the thrombus site in _Cy5.5‐Sr_EcN_PL_ group of mice, and it could be observed as early as 10 min. The fluorescence of the thrombus site in mice of _Cy5.5‐Sr_EcN_PL_ group gradually increased over time. Through slice analysis of LCCA blood vessel in different groups of mice at different administration times. The results (Figure 5j; Figure S18, Supporting Information) showed that the fluorescence intensities of Cy5.5‐rt‐PA and _Cy5.5‐Sr_EcN were similar, and the fluorescence intensity of group _Cy5.5‐Sr_EcN_PL_ was significantly stronger than that of both groups, which proved that PMV(L‐Arg) provided thrombus‐targeting ability for _Sr_EcN_PL_. In addition, a large number of probiotic aggregates could be clearly observed in _Cy5.5‐Sr_EcN_PL_ group of vascular sections (Figure S19, Supporting Information). The above results demonstrate that _Sr_EcN_PL_ has significant thrombus targeting ability and exhibits significant enrichment in thrombus tissue. The thrombus targeting effect of _Sr_EcN_PL_ originates from the modification of PMV(L‐Arg), which proves the rationality of this study for the design of modification to EcN for thrombus treatment.

### 2.6 Thrombolysis Efficacy Evaluation of _Sr_EcN_PL_ In Vivo

**Figure 6:**
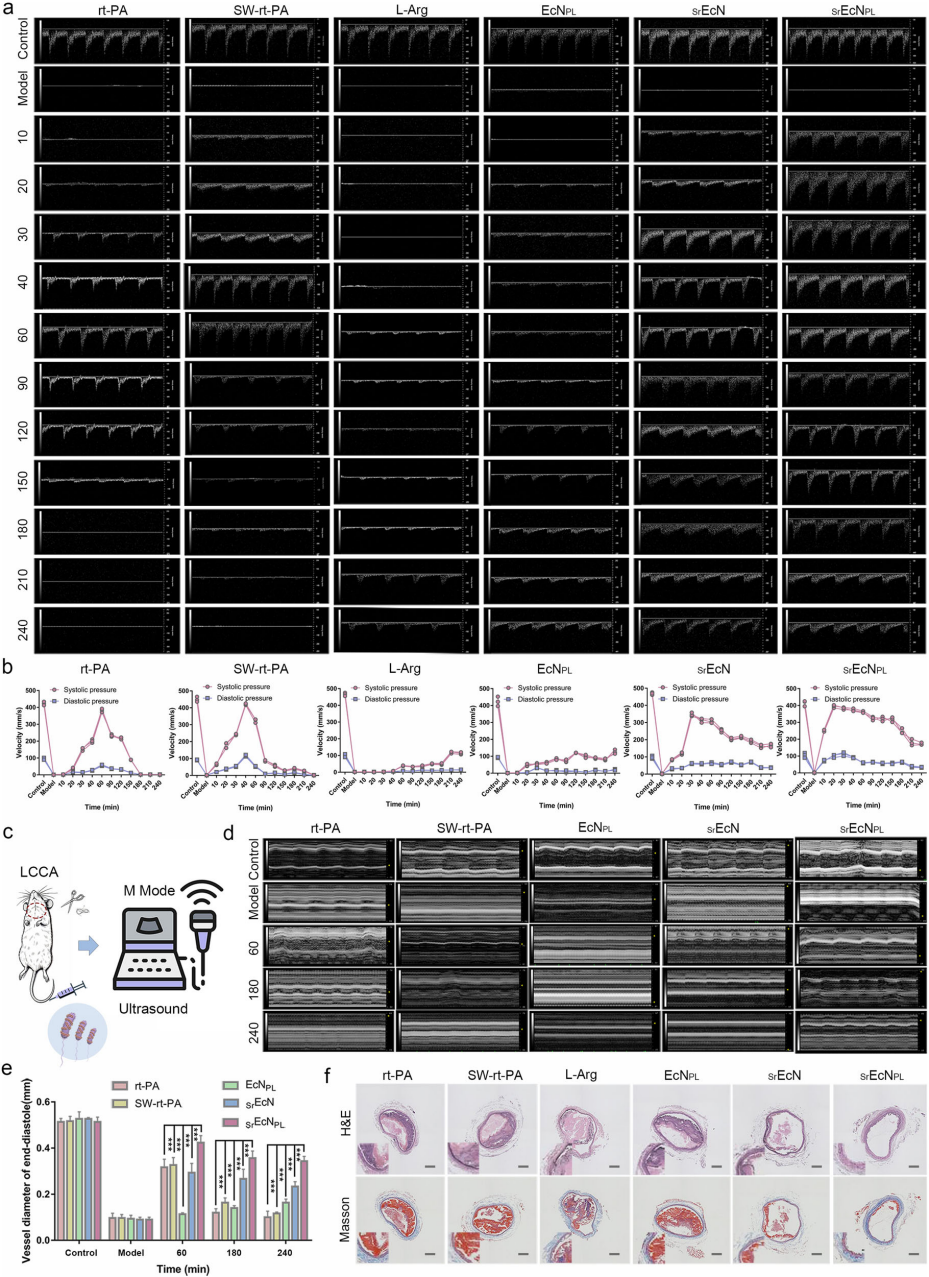
Thrombolysis efficacy investigation of the _Sr_EcN_PL_ robot in vivo. a,b) Doppler ultrasound analysis of blood velocity in different treatment groups (rt‐PA, SW‐rt‐PA, L‐Arg, EcN_PL_, _Sr_EcN, _Sr_EcN_PL_) in left common carotid artery thrombus model mice. (n = 3; mean ± SD). c) Experimental method for Doppler ultrasound analysis of vascular inner diameter. d,e) Ultrasound analysis of the inner diameter of blood vessels in mice across different treatment groups (rt‐PA, SW‐rt‐PA, _Sr_EcN, EcN_PL_ and _Sr_EcN_PL_) at various time poiunts. (n = 3; mean ± SD). ***P < 0.001. f) H&E and Masson staining analysis of blood vessels from different treatment groups (rt‐PA, SW‐rt‐PA, L‐Arg, EcN_PL_, _Sr_EcN, _Sr_EcN_PL_). Scale bars, 200 µm.

An in vivo LCCA thrombus model was constructed as the method described in section 1.5. Free allocation of thrombus model mice were randomly divided into six groups (rt‐PA, SW‐rt‐PA, L‐Arg, EcN_PL_, _Sr_EcN, _Sr_EcN_PL_, 1.5 × 10^4^ CFU mL^−1^, equal to 0.48 mg mL^−1^ rt‐PA and 0.23 mg mL^−1^ L‐Arg) with the dose of rt‐PA was 2 mg kg^−1^. Doppler ultrasound in PW mode was used to analyze the blood flow of LCCA vessels in different treatment groups of mice at different times. As shown in **Figure** **6**a,b, both rt‐PA and SW‐rt‐PA exhibited significant thrombolytic effects. The rt‐PA group achieved peak blood flow in ≈60 min, while the SW‐rt‐PA group reached it in ≈40 min. This difference might be attributed to the rod‐shaped structure of SW‐rt‐PA, which enhanced thrombus penetration, as well as the high drug concentration per unit surface area resulting from the extensive rt‐PA modification on the SWCNT surface. The thrombolysis efficiency of _Sr_EcN was faster than that of SW‐rt‐PA, indicating that EcN played an important role in drug delivery of rt‐PA. Through the autonomous movement and thrombus penetration of EcN, the thrombolysis time was shortened. However, both rt‐PA and SW‐rt‐PA showed blood flow obstruction at 180 min and 210 min respectively, resulting in secondary thrombus.

The L‐Arg and EcN_PL_ drugs showed no significant thrombolytic effect within 4 h. However, the thrombolytic time in groups EcN_PL_ and L‐Arg was ≈20 min earlier, possibly due to the modification of EcN and PMV(L‐Arg) enhancing the thrombus‐targeting mobility of L‐Arg. Although EcN_PL_ and L‐Arg did not show any secondary thrombus within 4 h, their thrombolytic performance was subpar. In contrast, _Sr_EcN_PL_ showed a significant thrombolytic effect within 10 min, 20 min faster than _Sr_EcN likely due to the modification of PMV(L‐Arg) increasing thrombus targeting of _Sr_EcN_PL_. Both _Sr_EcN and _Sr_EcN_PL_ showed good thrombolytic effects and no secondary thrombus within 4 h. Among the six treatments, _Sr_EcN_PL_ showed the fastest thrombolytic effect and sustained thrombolysis within 4 h, exhibiting excellent thrombolytic effects in vivo.

The ultrasound blood flow analysis results showed that L‐Arg had no significant thrombolytic effect. In order to verify its rationality as a vasodilator drug designed as a modification of EcN, ultrasound M‐mode analysis was used to assess vascular inner diameter changes. The experimental method for Doppler ultrasound analysis of vascular inner diameter was shown in Figure 6c, where the vascular diameter changes in LCCA thrombus model mice after administration of drugs were analyzed by ultrasound M‐mode. As shown in Figures 6d,e, and S20 (Supporting Information), the vascular diameters of rt‐PA, SW‐rt‐PA, _Sr_EcN and _Sr_EcN_PL_ groups significantly increased compared to the model group at 60 min, which may be due to the thrombolytic effect of rt‐PA. However, the vascular diameters of rt‐PA and SW‐rt‐PA groups significantly decreased at 180 min, possibly due to the formation of secondary thrombus that blocked the blood vessels. The blood vessels diameters of EcN_PL_ increased over time, attributed to the vasodilatory effect of L‐Arg and the rapid thrombus targeting movement of EcN_PL_. The _Sr_EcN_PL_ group showed a significant and sustained increase in vascular diameter, higher than the _Sr_EcN group, due to the combined vasodilatory effect of L‐Arg and the thrombolytic effect of rt‐PA.

To confirm whether the vasodilator effects of _Sr_EcN_PL_ in vivo could be attributed to its L‐Arg payload, blood vessel tissue from the thrombus site in mice were excised and sectioned after 3 h of treatment. The treatment groups were as follows: saline, L‐Arg, EcN_PL_, and _Sr_EcN_PL_ (1.5 × 10⁴ CFU mL^−1^, equivalent to 0.48 mg mL^−1^ rt‐PA and 0.23 mg mL^−1^ L‐Arg), with rt‐PA administered at a dose of 2 mg kg^−1^. As shown in Figure S21 (Supporting Information), slices from different groups were stained using a NO staining kit. The fluorescence intensity in the L‐Arg group increased significantly compared to the saline group, but was significantly lower than that in EcN_PL_ group. This difference was likely due to the significant thrombus fast movement‐targeting ability of EcN_PL_. The fluorescence intensity of _Sr_EcN_PL_ was comparable to that of EcN_PL_ (Figure S22, Supporting Information), indicated that _Sr_EcN_PL_ also produced substantial NO, demonstrated its significant vasodilation ability. H&E and Masson staining analysis of blood vessels from different treatment groups (rt‐PA, SW‐rt‐PA, L‐Arg, EcN_PL_, _Sr_EcN, _Sr_EcN_PL_) after 4 h of treatment were shown in Figures 6f and S23 (Supporting Information). The thrombus therapeutic effect of _Sr_EcN was stronger than that of rt‐PA, SW‐rt‐PA, L‐Arg and EcN_PL_. Moreover, the thrombus therapeutic effect of _Sr_EcN_PL_ was stronger than _Sr_EcN, proving that the combination of rt‐PA and L‐Arg offered the best thrombus therapeutic effect and inhibited the formation of secondary thrombus. The above results demonstrate that the _Sr_EcN_PL_ robot constructed in this study has ideal thrombolytic therapy effects and significant inhibitory effects on secondary thrombus.

In order to investigate the biological safety of _Sr_EcN_PL_ robot, the hemorrhagic time, blood routine, blood biochemistry, and pathological sections of major organs in mice treated with (saline, rt‐PA, SW‐rt‐PA, _Sr_EcN, _Sr_EcN_PL_, 1.5 × 10^4^ CFU mL^−1^, equal to 0.48 mg mL^−1^ rt‐PA) with the dose of rt‐PA is 2 mg kg^−1^ were analyzed. As shown in Figure S24 (Supporting Information), the hemorrhagic time of rt‐PA was the longest compared to the other four groups, indicated sever hemorrhagic side effects. The hemorrhagic time of SW‐rt‐PA and _Sr_EcN were slightly shorter than that of _Sr_EcN_PL_, possibly due to their faster thrombolytic effect and faster consumption of thrombolytic drugs carried. Compared with rt‐PA, SW‐rt‐PA, and _Sr_EcN, _Sr_EcN_PL_ had the shortest hemorrhage time, attributed to its thrombus targeting properties, which reduced the accumulation of thrombolytic drugs in non‐target tissues, thereby reduced the occurrence of hemorrhagic side effects. These results demonstrate that _Sr_EcN_PL_ not only prolongs the biological half‐life of rt‐PA but also markedly decreases its hemorrhagic complications, validating both the mechanistic rationale and clinical potential of our thrombolytic strategy.

In order to verify the biological safety of administering large quantities of probiotics intravenously into the bloodstream, the blood routine and biochemical results of mice in different treatment groups (saline, EcN, _Sr_EcN_PL_, 1.5 × 10^4^ CFU mL^−1^) were analyzed. The blood routine results as shown in Figure S25 (Supporting Information), the blood routine parameters of the _Sr_EcN_PL_ were comparable to the control group, with no significant difference. However, some parameters of EcN showed significant differences compared to the control group, such as LYM#, LYM%, MONO#, MONO%, PLT and WBC decreased, while NEUT% increased. The changes in these parameters indicated an inflammatory response in the mice due to probiotic infection. The biochemical results (Figure S26, Supporting Information) revealed similar trends, with the _Sr_EcN_PL_ biochemical parameters closely matching the control group, while EcN‐treated mice exhibited elevated levels of ALT, AST, LDH, CK, CK‐MB, CREA and UA. The changes in these parameters revealed that mice suffer from hepatitis and nephritis. H&E staining analysis was performed on the main organs of mice in different treatment groups. As shown in the Figure S27 (Supporting Information), the liver and kidney tissues of the EcN group exhibited inflammatory cell infiltration, showing pathological features of hepatitis and nephritis. These findings were consistent with the results of Figure S26 (Supporting Information). However, the _Sr_EcN_PL_ treatment group showed no significant damage to any major organs. The above results indicate that unmodified probiotics can exhibit biological toxicity when administered intravenously, but surface modification of probiotics can significantly reduce their biological toxicity, potentially by inhibiting their reproduction.

## 3 Conclusion

In summary, we report an engineered probiotic driven micro‐rod robot. This robotic system employs natural probiotics as biohybrid carriers, functionally augmented with: thrombolytic carbon nanotubes and platelet membrane‐coated nanoparticles loaded with vasodilators. This dual‐modification enables targeted thrombus therapy through synergistic mechanisms. This robot based on the amazing autonomous movement speed of low immunogenicity EcN, combined with the active targeting performance of platelet membrane to deliver thrombolytic drugs quickly and accurately to thrombus tissue. Its natural rod‐shaped structure allows for deeper penetration into thrombus tissue compared with sphere particles, enabling the thrombus to disintegrate from the inside and achieving faster thrombolysis efficiency.

In order to suppress the formation of secondary thrombus as much as possible, this study combines the vasodilator L‐Arg with the thrombolytic drug rt‐PA. By utilizing the in vivo long circulation effect of thrombolysis micro‐robots and the vasodilation and repair effect of L‐Arg, the blood flow is maintained for a long time, thereby inhibiting the formation of secondary thrombus. The in vivo and in vitro research results indicate that this micro‐robot can rapidly and accurately target and penetrate thrombus tissue, prolong the biological half‐life of thrombolytic drugs by ≈332 times, and achieve sustained thrombolysis while reducing secondary thrombus formation.

This novel dual‐pronged thrombolytic therapy is of great scientific significance for treating vascular injury induced thrombotic diseases and those with narrow therapeutic windows. In addition, this study reveals that unmodified probiotics exhibit certain biological toxicities, including blood inflammation and mild hepatic and renal inflammation. The probiotic modification method used in this study successfully reduces these biological toxicities, providing valuable insights for future probiotic modification strategies.

## 4 Experimental Section

### Prepare and Characterization of EcN and SW‐rt‐PA

First, a large amount of highly active *Escherichia coli Nissle 1917* was cultured. The EcN was obtained from BeNa Culture Collection (BNCC361741). The bacteria were cultured in Luria‐Bertani (LB, BNCC372161) broth and grown to the logarithmic phase with continuous shake at 220 rpm at 37 °C.

The synthesis steps of thrombolytic carbon nanotubes SW‐rt‐PA were as follows: The highly purity carboxylate single‐walled carbon nanotubes (SW‐COOH) were purchased from Nanjing/Jiangsu XFNANO Materials Tech Co., Ltd (101 369, Purity greater than 95%, diameter 1–2 nm, length 1–3 µm). The SW‐COOH underwent three freeze‐thaw cycles using liquid nitrogen and a 37 °C‐water bath, following the same procedure as for platelet membrane extraction. Then, the surface of SW‐COOH was modified with the protein drug rt‐PA. Briefly, 2.0 mg of carbonylated single‐walled carbon nanotubes were suspended in 2.0 mL of 100 mM MES buffer solution and subjected to 20 min of ultrasonic treatment (100 w, 30% power) under ice bath condition. Next, 1 mL of NHS aqueous solution (50 mg/mL^−1^) and 1 mL of fresh EDAC aqueous solution (50 mg mL^−1^) were quickly added under rapid stirring. The mixed solution was stirred at room temperature for 30 min. Then, add 1 mL (10 mg mL^−1^) of rt‐PA (alteplase, MedChemExpress (Monmouth Junction, NJ, USA), HY‐108865) in 50 mM MES buffer solution (10 mg mL^−1^) was added to the mixed solution, and the reaction solution was stirred overnight at 4 °C. Afterwards, the reaction solution was washed three times with 50 mM MES buffer solution by centrifugation (10 000 rpm, 10 min) to remove unused catalyst and protein. After washing, the reaction solution was filtered through a 0.45 µm filter membrane to obtain thrombolytic carbon nanotubes with a smaller particle size. The thrombolytic carbon nanotube SW‐rt‐PA was dispersed in PBS and stored at 4 °C.

TEM, SEM and AFM microscopy were used to analyze the particle size and morphology of SW‐COOH and SW‐rt‐PA. The encapsulation efficiency (EE) and drug loading (DL) of rt‐PA on SW‐COOH were analyzed by protein quantification method using a BCA protein assay kit (YEASEN Biotechnology (Shanghai) Co., Ltd). The EE% was calculated as (Amount of rt‐PA bound to SW‐COOH/Total amount of rt‐PA added) ×100%. The DL% (after filter) was calculated as (Amount of rt‐PA bound to SW‐COOH/Total weight of SW‐rt‐PA) ×100%. The amount of protein bound to SW‐COOH was calculated by the equation: amount of protein bound = total protein amount – free protein amount. The total weight of SW‐rt‐PA was determined from the weight of the freeze‐dried SW‐rt‐PA product.

### Extraction and Characterization of PMV(L‐Arg)

First, platelets were extracted and purified from peripheral blood using the following protocol: After anaesthetizing C57BL/6J strain mice with isoflurane, blood was collected from the eyeballs^[^ ^63^, ^64^ ^]^ and placed in anticoagulant tubes. Two milliliters of fresh anticoagulant were diluted with an equal volume of 10 mM PBS. Platelets were separated from the blood by a human peripheral blood platelet separation kit (P6390, Beijing Solarbio Science & Technology Co., Ltd.). Briefly, 4 mL of platelet separation solution was added to a centrifuge tube, followed by 2 mL of 60% separation solution (1200 µL platelet separation solution + 800 µL tissue dilution solution) were added to form a separation interface. Then, 4 mL of diluted anticoagulant whole blood (prepared within 2 h) were added, and the tube was centrifuged at room temperature for 20 min at a speed of 350 g. After centrifugation, the platelet layer (indicated by a red box in Figure 1a) was carefully aspirated into a 15 mL clean centrifuge tube, and 10 mL of PBS were added, followed by centrifugation at 500 g for 20 min. After centrifugation, the supernatant was removed, and 1 mL of PBS was added to obtain a platelet suspension.

Second, platelet membranes were extracted and purified from the platelet suspension. One milliliter of platelet suspension was added into a 2 mL cryopreservation tube. The cryopreservation tube was placed in liquid nitrogen for 10 min, then immediately transferred to a 37 °C‐water bath. After ≈15 min, the cryopreservation tube was removed and restored at room temperature. This freeze‐thaw process was repeated three times as described above. After the final thaw, the ruptured platelet suspension was further centrifuged (2000 g, 5 min) to prepare platelet membrane vesicles. The obtained platelet membrane vesicles (PMV) were resuspended in 10 mM PBS and washed three times with PBS containing protease inhibitors by centrifugation (2000 g, 5 min) to separate platelet membranes and their organelles, resulting in purified platelet membranes.

Finally, L‐Arg was loaded into the platelet membranes to prepare platelet membrane drug‐loaded nanoparticles PMV(L‐Arg). The purified platelet membrane vesicles were resuspended in L‐Arginine (50 mM) solution (PMV/L‐Arg = 15:1) and subjected to 15 min of ultrasonic treatment (100 w, 20% power) under ice bath conditions. An Avestin micro extruder was used to extrude the mixed liquid through a 200 nm polycarbonate porous membrane 10 times to obtain platelet membrane drug‐loaded nanoparticles. Unloaded L‐Arg drugs was removed via dialysis method (2000 Da, dialysis bag aperture), and the harvested drug‐loaded platelet membrane vesicles PMV(L‐Arg) were stored in PBS at 4 °C.

The morphology of platelets, platelet membranes, platelet membrane vesicles and PMV(L‐Arg) nanoparticles were analyzed using TEM, SEM and CLSM microscopy. DLS was used to analyze the particle size and zeta potential of PMV(L‐Arg) nanoparticles.^[^ ^65^, ^66^ ^]^ Flow cytometry and CLSM were used to analyze the expression of platelet characteristic proteins CD61 and CD41. The entrapment efficiency rate and drug‐loading rate of L‐Arg from platelet membrane vesicles were measured using an ultraviolet spectrophotometer with an absorption wavelength of 205 nm. EE% was calculated as (Amount of L‐Arg loaded in PMV/Total amount of L‐Arg added) ×100%, and DL% (after filter) was calculated as (Amount of L‐Arg loaded in PMV/Total weight of PMV(L‐Arg)) ×100%. The amount of L‐Arg loaded in PMV was determined by the equation: amount of L‐Arg loaded in PMV = total L‐Arg amount – free L‐Arg amount. The total weight of PMV(L‐Arg) was obtained from the weight of the freeze‐dried PMV(L‐Arg).

### Prepare and Characterization of _Sr_EcN_PL_

The engineered probiotic driven micro‐rod robot _Sr_EcN_PL_ was prepared as follows: First, EcN and thrombolytic carbon nanotube SW‐rt‐PA were prepared as described above. Then, SW‐rt‐PA was modified on the surface of EcN to obtain _Sr_EcN. Briefly, 2.0 mg of SW‐rt‐PA was suspended in 2.0 mL of 100 mM MES buffer solution. Under rapid stirring, 1 mL of NHS aqueous solution (50 mg/mL) and 1 mL of fresh EDAC aqueous solution (50 mg mL^−1^) were quickly added. The mixed solution was stirred at room temperature for 30 min. Then, 1 mL (10^4^ CFU) of EcN LB culture medium was added to the mixed solution, and the reaction solution was stirred for 3 h at 4 °C. Afterwards, the reaction solution was washed three times with LB culture medium by centrifugation (3000 rpm, 10 min) to remove unbound SW‐rt‐PA, yielding _Sr_EcN.

Subsequently, PMV(L‐Arg) nanoparticles were modified on the surface of _Sr_EcN. First, Sulfo‐NHS‐Biotin (500 µM) and streptavidin (500 µM) were prepared in 10 mM PBS. PMV(L‐Arg) nanoparticles derived from 2 mL of blood were mixed with Sulfo‐NHS‐Biotin (4 mL, 500 µM) and stirred at room temperature for 1 h. After centrifugation at 12 000 g for 10 min, unreacted Sulfo‐NHS‐Biotin was removed, and biotin‐PMV(L‐Arg) was obtained. A 2 mL aliquot of _Sr_EcN bacterial solution (10^4^ CFU) was incubated at 37 °C. Then, 1 mL of Sulfo‐NHS‐Biotin (4 mL, 500 µM) and 1 mL of streptavidin (4 mL, 500 µM) were added and incubated for 1 h each. After centrifugation at 3000 rpm for 10 min, the pellet was resuspended in LB broth medium to obtain _Sr_EcN‐biotin‐streptavidin. _Sr_EcN‐biotin‐streptavidin was mixed with biotin‐SW‐rt‐PA, incubate at 37 °C for 1 h, and then centrifuged (3000 rpm, 10 min). The obtained _Sr_EcN‐biotin‐streptavidin‐biotin‐PMV(L‐Arg), designated as the engineered probiotic driven micro‐rod robot _Sr_EcN_PL_, was resuspended in LB broth medium and stored at 4 °C.

The particle size and morphology of _Sr_EcN_PL_ were analyzed using TEM and SEM microscopy. For CLSM analysis of _Sr_EcN_PL_, three different components were stained as follows: Cy5.5‐NHS (10 µM) was used to modify rt‐PA proteins to prepare SW‐rt‐PA‐Cy5.5, and the cell membrane staining probe DiR (5 µM, TargetMol, USA; TD0085) was used to stain platelet membranes to prepare DiR‐PMV(L‐Arg) with preparation steps as described above. SW‐rt‐PA‐Cy5.5 and DiR‐PMV(L‐Arg) were surface‐modified onto EcN to obtain _Cy5.5‐Sr_EcN_,_ EcN_PL‐DiR,_ and _Cy5.5‐Sr_EcN_PL‐DiR_, with EcN stained using the live probiotic staining probe SYTO9 (5 µM). Sodium dodecyl sulfate‐Polyacrylamide gel electrophoresis (SDS‐PAGE, P1250 M, Beijing LABLEAD Inc.) was used to separate and detect the proteins in different components (rt‐PA, SW‐rt‐PA, PMV(L‐Arg), EcN, EcN_PL_, _Sr_EcN, _Sr_EcN_PL_, Marker). The activity of rt‐PA was measured with the chromogenic subtract S‐2288 (Chromogenix, no. 82 085 239). The kinetic activity of rt‐PA in SW‐rt‐PA and _Sr_EcN_PL_ was determined by mixing SW‐rt‐PA and _Sr_EcN_PL_ PBS solution with 1 mM assay buffer (S‐2288 in 0.1 mM Tris‐HCl pH 8.4) in a 96‐well cell culture plate. Absorbance at 405 nm was recorded, and the kinetic activity η was determined as the linear slop of ΔA = A(t) − A(0) versus test time t, η = ΔAt. The activity retention of _Sr_EcN_PL_ was determined as the ratio of their kinetic activity to the kinetic activity of free rt‐PA solution at the same concentration.

To evaluate the EE and DL of SW‐rt‐PA and PMV(L‐Arg) in _Sr_EcN_PL_, SW‐rt‐PA‐Cy5.5 and DiR‐PMV(L‐Arg) were prepared to obtain _Cy5.5‐Sr_EcN_PL‐DiR_, with EcN stained using the probe SYTO9. Fluorescence spectrophotometry was used to measure the fluorescence intensity of SW‐rt‐PA‐Cy5.5 and DiR‐PMV(L‐Arg) before and after modification. EE% (SW‐rt‐PA or PMV(L‐Arg)) was calculated as (fluorescence intensity of Cy5.5 or DiR after modification/fluorescence intensity of Cy5.5 or DiR before modification) ×100%. DL% was determined by comparing the fluorescence intensities of Cy5.5 or DiR with that of Cy5.5+DiR+SYTO9 in _Cy5.5‐Sr_EcN‐SYTO9_PL‐DiR_, using the following equation: DL% (SW‐rt‐PA or PMV(L‐Arg)) = (fluorescence intensity of Cy5.5 or DiR/ fluorescence intensity of Cy5.5+DiR+SYTO9) ×100%.

### Probiotic Activity Analysis of _Sr_EcN_PL_

The probiotic activity of _Sr_EcN_PL_, before and after surface modification of SW‐rt‐PA and PMV(L‐Arg), was analyzed using laser confocal microscopy (Leica SP8, 100×) and flow cytometry. Live bacteria and dead bacteria were stained with SYTO9 and PI probes in equal proportions, respectively. The excitation and emission wavelengths of SYTO9 and PI were 480 nm/500 nm and 490 nm/635 nm, respectively. A laser confocal microscopy (Leica SP8, 63×) was used to observe the live and dead bacteria of EcN and _Sr_EcN_PL_ in different times (1 h, 3 h and 6 h). A UV spectrophotometer was used to measure the OD values of different bacterial solutions (EcN, _Sr_EcN_PL_) at an absorbance wavelength of 600 nm.

### The Motion Behaviors Evaluate of _Sr_EcN_PL_

To investigate the motility of EcN modified with SW‐rt‐PA and PMV(L‐Arg), laser confocal microscopy (Leica SP8, 100×) was used to observe the motility before and after modification. The movement videos of EcN and _Sr_EcN_PL_ (1.5×10^4^ CFU/mL) in serum were shown in Movie S1 (Supporting Information), the motion trajectory and coordinates from video were analyzed by Image J software. MATLAB software with operation the code written by the research group were used to calculate MSD value, motion velocity, and Deff value based on the coordinate data of particle motion trajectory. The MSD was calculated using the following equation, MSD (Δ*t*) = 4 *D* Δ*t* + *v* ^2^Δ*t* ^2^, where *D* is the diffusion coefficient, *t* is the time, and *v* is the motion velocity.^[^ ^22^ ^]^

### Cumulative Release of L‐Arg from _Sr_EcN_PL_

Dialysis method^[^ ^67^, ^68^, ^69^ ^]^ was used to investigate the L‐Arg and rt‐PA drug release from _Sr_EcN_PL_. Briefly, L‐Arg (1 mM) and _Sr_EcN_PL_ (1 × 10^4^ CFU) were placed in equal proportions into a dialysis bag (1000 Da) and immersed in a solution medium either with or without PLA2 (200 ng mL^−1^). The dialysis bag was then placed in a shaker and shaken at room temperature for 48 h. At predetermined time intervals, 20 µL samples of the different solutions (L‐Arg‐PLA2, L‐Arg+PLA2, _Sr_EcN_PL_‐PLA2, _Sr_EcN_PL_+PLA2,) were withdrawn from the dialysis bag. A UV spectrophotometer (absorption wavelength: 205 nm) and a human rt‐PA ELISA kit (Shanghai Jianglai Biology Co, Ltd. JL14148‐48T) were used to analyze the samples at different time points (1, 2, 4, 6, 8, 10, 12, 24, and 48 h).

### In Vitro Target Analysis of _Sr_EcN_PL_

To investigate the platelet targeting ability of _Sr_EcN_PL_, a Transwell chamber (0.45 µm) was used. Purified platelet cells (labeled by DiR probe with 5 µM) were cultured in the lower chamber. Meanwhile, 0.2 mL of different bacterial solutions (EcN, _Sr_EcN, EcN_PL_, _Sr_EcN_PL_, 1.5 × 10^4^ CFU mL^−1^, equal to 0.48 mg mL^−1^ rt‐PA, 0.23 mg mL^−1^ L‐Arg) were added to the upper chamber in equal colony counts. After a 3 h incubation, the upper bacterial solution was aspirated, and the upper chamber was removed. The live bacterial staining probe SYTO9 (5 µM) was used to stain EcN from different groups present in the lower chamber. The samples were then observed under a confocal laser microscope with excitation and emission wavelengths of 480 nm/500 nm and 748 nm/780 nm. The Image J software was used to analyze the fluorescence intensity and fluorescence co‐localization of the different groups.

### In Vitro Thrombolysis Experiment Analysis of _Sr_EcN_PL_

In order to investigate the in vitro thrombolytic effect of _Sr_EcN_PL_ robot, an equal volume of fresh blood was loaded into a syringe tube and divided into 5 groups. Each group was treated with the same concentration of rt‐PA and the same volume (200 µL) of samples (PBS, rt‐PA, SW‐rt‐PA, _Sr_EcN, _Sr_EcN_PL_, 1.5 × 10^4^ CFU mL^−1^, equal to 0.48 mg mL^−1^ rt‐PA). The samples were left to stand at room temperature for 12 h, with solid‐liquid separation performed hourly. The volume of liquid blood obtained from thrombolysis was recorded, and statistical analysis was conducted using GraphPad software (version 9.0).

To evaluate the thrombus penetration ability of _Sr_EcN_PL_ robot, EcN was stained with the probe SYTO9 (5 µM). Thrombus blocks from the syringe of different groups of probiotics (EcN, _Sr_EcN, _Sr_EcN_PL_, 1.5 × 10^4^ CFU mL^−1^) were removed at different time points as previously described. These thrombus blocks were then frozen, cross‐sectioned, and observed under a fluorescence microscope (20×) at different time points (1, 3, and 6 h). The penetration depth of the probiotics in each group was recorded and analyzed using Photoshop software (2022 version) and GraphPad software (version 9.0).

### Construction of Thrombus Model In Vivo

Based on previous research by the research group, a in vivo left carotid artery thrombus model was constructed using the ferric chloride induction method.^[^ ^22^ ^]^ The specific process is as follows: C57BL/6 mice (18 ± 2 g, 8 weeks old) were provided with free access to food and drinking water.^[^ ^70^, ^71^ ^]^ Under isoflurane anesthesia conditions, the mice's necks were shaved, and the skin was incised with surgical scissors to expose the left common carotid artery after removing surrounding fat and connective tissue. A filter paper (3 × 1 mm) soaked in 10% FeCl_3_ solution was placed on the surface of the left common carotid artery bifurcation in mice for 5 min and then removed. After ≈10 min, a distinct yellow clot could be observed under a surgical microscope. The wound was sutured, and ultrasound analysis (B Mode, Color Mode, PW Mode, and M Mode) was performed on the mice's necks before and after thrombus formation. After that, the left common carotid artery bifurcation vessels before and after thrombus model were taken out for sectioning and H&E staining.

### Pharmacokinetic Evaluation of _Sr_EcN_PL_ in vivo

SD rat (250 ± 10 g, n = 5) were provided with free access to food and drinking water. A mouse left common carotid artery thrombus model was constructed as above, and the rats were divided into two groups (rt‐PA, _Sr_EcN_PL_, 1.5 × 10^4^ CFU mL^−1^, equal to 0.48 mg mL^−1^ rt‐PA) freely. Cy5.5 was used to label rt‐PA, and the prepared _Cy5.5‐Sr_EcN_PL_ was administered via tail vein injection. At different time points (0, 0.083, 0.17, 0.33, 0.5, 1, 2, 3, 4, 6, 8, 12, 24, and 48 h), 500 µL of mouse orbital venous blood was collected using glass tubes with 0.45 µm aperture and transferred to EDTA anticoagulant tube.^[^ ^67^ ^]^ The blood samples were centrifuged (3000 rpm, 10 min), and the fluorescence intensity of the supernatant was analyzed using a fluorescence spectrophotometer at excitation and emission wavelengths of 673 and 695 nm, respectively. Different pharmacokinetic parameters of two groups were calculated and analyzed by DAS software (version 2.0).

### Thrombus Targeting Analysis of _Sr_EcN_PL_ In Vivo

First, a mouse left common carotid artery thrombus model was constructed as described above, and the mice were divided into three groups (n = 3). The Cy5.5 probe was used to label rt‐PA, preparing Cy5.5‐rt‐PA, _Cy5.5‐Sr_EcN and _Cy5.5‐Sr_EcN_PL_ (1.5 × 10^4^ CFU mL^−1^, equal to 0.48 mg mL^−1^ rt‐PA) for tail vein administration in mice with the dose of rt‐PA 2 mg kg^−1^. At different time points (0, 10, 20, 30, 40 and 60 min), the three groups of model mice were placed in a small animal fluorescence imaging device for observation. The fluorescence intensity at the thrombus sites in different groups of mice was recorded at different time points using Image J software. The left carotid artery blood vessels of different groups of mice were harvested, frozen, sectioned, and observed different vessels slices under a fluorescence microscope at time points 30 and 60 min.

### Thrombolysis Efficacy Evaluation of _Sr_EcN_PL_ In Vivo

A left common carotid artery thrombus model was constructed in C57BL/6 mice (18 ± 2 g, 8 weeks old, n = 5), which were then divided into 6 groups (rt‐PA, SW‐rt‐PA, L‐Arg, EcN_PL_, _Sr_EcN, _Sr_EcN_PL_, 1.5 × 10^4^ CFU mL^−1^, equal to 0.48 mg mL^−1^ rt‐PA) with an rt‐PA dose of 2 mg kg^−1^. Doppler ultrasound in PW mode was used to analyze the blood flow in the left common carotid artery of mice from different groups before and after thrombus induction and at various times after tail vein administration (control, model, 10, 20, 30, 40, 60, 90, 120, 150, 180, 210 and 240 min). GraphPad software (version 9.0) was used for statistical analysis of blood flow from different groups.

In order to validate its rationality as a vasodilator drug designed as a modification of EcN, ultrasound M‐mode analysis of vascular inner diameter was performed on mice from different treatment groups at different time points (control, model, 60, 180 and 240 min). To demonstrate that the vasodilation effect originates from L‐Arg, the specific NO content produced by L‐Arg of _Sr_EcN_PL_ was assessed. After 3 h of treatment, blood vessel tissue from the thrombus site was excised and sectioned from mice in different treatment groups (saline, L‐Arg, EcN_PL_, _Sr_EcN_PL_, 1.5 × 10^4^ CFU mL^−1^, equal to 0.48 mg mL^−1^ rt‐PA, 0.23 mg mL^−1^ L‐Arg) with the dose of rt‐PA 2 mg kg^−1^. Tissue sections were stained using a NO staining kit, and the fluorescence intensity in different sections was observed via fluorescence microscopy. H&E and Masson staining were used to analyze of blood vessels from different treatment groups (rt‐PA, SW‐rt‐PA, L‐Arg, EcN_PL_, _Sr_EcN, _Sr_EcN_PL_) after 4 h of treatment.

### Safety Evaluation of _Sr_EcN_PL_ In Vivo

In order to investigate the biological safety of _Sr_EcN_PL_ robot, the hemorrhagic time, blood routine, blood biochemistry, and pathological sections of major organs were conducted in mice treated with _Sr_EcN_PL_ robot (1.5×10^4^ CFU/mL).

Hemorrhagic time experiment: An in vivo thrombus model was established as previously described. Mice were administered via vein injection with different agents (saline, rt‐PA, SW‐rt‐PA, EcN_PL_, _Sr_EcN_PL_, 1.5 × 10^4^ CFU mL^−1^, equal to 0.48 mg mL^−1^ rt‐PA) at an rt‐PA dose of 2 mg kg^−1^. Immediately after administration, the tails of the mice were cut by 1 centimeter using surgical scissors, and the cut ends were immersed in physiological saline at 37 °C. The time from the onset of hemorrhage to the cessation of hemorrhage was recorded as the hemorrhagic time. The hemorrhagic time for each group was analyzed using GraphPad software (version 9.0).

Blood routine, blood biochemistry and pathology experiment: The in vivo thrombus model was constructed as mentioned above. After 12 h of tail vein administration of different agents (saline, EcN, _Sr_EcN_PL_, 1.5 × 10^4^ CFU mL^−1^, equal to 0.48 mg mL^−1^ rt‐PA) at an rt‐PA dose of 2 mg kg^−1^, blood samples and main organs of mice from different groups were collected. Blood routine and blood biochemistry test kits were used to detect parameter changes in different blood samples. The main organs (heart, liver, spleen, lung, and kidney) of the mice stained by H&E staining kit and Masson staining kit, and the histological changes in different organs were observed under a microscope.

### Statistical Analysis

The raw data were normalized, and the results are presented as mean ± SD (n ≥ 3 biological replicates). Error bars indicate the standard deviation of independent experimental replicates. Statistical comparisons were performed by two‐sided unpaired t‐tests, with significance levels defined as follows: *P < 0.05, **P < 0.01, and ***P < 0.001. All statistical analyses were performed by GraphPad Prism software (version 9.0).

### Ethics Approval

All the animal experiments performed with the approval of the Xiamen University Experimental Animal Center and all procedures performed according to the guidelines and animal welfare protocols. Animal ethics number of Xiamen university animal experiment center was XMULAC20220249.

## Conflict of Interest

The authors declare no conflict of interest.

## Supporting information



Supporting Information

Supporting Information

Supporting Information

Supporting Information

Supporting Information

Supporting Information

## Data Availability

Research data are not shared.
