# Supplementary material for: Engineered Probiotic‐Powered Micro‐Rod Robot Bomb for Thrombus‐Penetrating Explosion
Source: Adv Sci (Weinh). 2025 Aug 28;12(36):e01304. doi: 10.1002/advs.202501304 (PMC12463029; doi:10.1002/advs.202501304)
Supplement: Supplementary file 1 — Supporting Information [file ADVS-12-e01304-s006.docx]

<Supporting information>

**Engineered Probiotic-Powered Micro-Rod Robot Bomb for Thrombus-Penetrating Explosion**

Riyue Liu, Jiale Chen, Yuanyuan Li, Nawsherwan, Cuilian Dai, Yan Wang, Jinrong Zheng *, Kecheng Zhou*, Mangmang Sang*

Prof. R.Y. Liu, Mr. J.L. Chen, Miss. Y.Y. Li, Prof. Nawsherwan, Prof. C.L. Dai, Prof. Y. Wang, Prof. J.R. Zheng, Prof. M.M. Sang

Xiamen Cardiovascular Hospital of Xiamen University,

School of Medicine,

Fujian Branch of National Clinical Research Center for Cardiovascular Diseases,

Xiamen, China.

E-mail: sangmangmang@xmu.edu.cn, [zhengjinrong@hebmu.edu.cn](mailto:%20Jinrong%20Zheng@xmu.edu.cn%20) , zhou_kc@126.com

Prof. K. C. Zhou, Miss. Y.Y. Li, Mr. J.L. Chen

Rehabilitation Medicine Center,

The Second Affiliated Hospital and Yuying Children's Hospital of Wenzhou Medical University, School of Rehabilitation Medicine, Wenzhou Medical University.

Wenzhou, Zhejiang, 325035, China.

School of Rehabilitation Medicine, Wenzhou Medical University, Wenzhou, Zhejiang, China.

Prof. J.R. Zheng

Hebei Key Laboratory of Cardiac Injury Repair Mechanism Study, The First Hospital of Hebei Medical University, Shijiazhuang 050031, China.

- 1. **Materials and instruments**

Brooke hysitron PI 95 transmission electron microscope test system and Zeiss sigma series field emission scanning electron microscope were used to analyze the morphology of nanoparticles. Leica SP8 laser confocal microscope was used to analysis the Motion Behaviors, celluer uptake and *in vivo* efficacy evaluation of micro-robot. BD FACS flow cytometry was used to analysis the platelet cell protein expression and bacterial activity. TissueFAXS SPECTRA (TissueGnostics, Austria) was used to panoramic scan of tissue section. IVIS® Spectrum (PerkinElmer, Austria) was used to *in vivo* imaging of mice. Ultra high resolution small animal ultrasound imaging system vevo2100 was used for the animal vascular function analysis.

- 1. **Biochemicals and Animals**

Alteplase was bought from MedChemExpress (MCE). L-Arg was bought from Fount Beijing Bio-Tech Co., LTD. Antibody CD41, antibody CD61 were brought from abcam. Sulfo NHS biotin, streptavidin were obatined from Thermo Fisher Technology (China) Co., Ltd. Cy5.5-NHS ester was obtained from Lumiprobe. Protein prefabricated adhesive purchased from Beijing LABLEAD Inc. H&E staining kit, masson staining kit, oil red O staining kit and reagents for cell culture were brought from Beyotime Biotechnology.

All animal procedures were performed in accordance with the guidelines for care and use of laboratory animals of Xiamen University and experiments were approved by the animal ethics committee of Xiamen University. Animal ethics number of xiamen university animal experiment center is XMULAC20220249.

**Table of abbreviations:**

| abbreviation | Full name |
| --- | --- |
| EcN | Escherichia coli Nissle 1917 |
| SWCNT-COOH | Carboxylated single-walled carbon nanotubes |
| EDC | 1,3-Dicyclohexylcarbodiimide |
| NHS | N-hydroxysuccinimide |
| PMV | Platelet membrane vesicle |
| MSD | mean-squared displacement |
| Δt | time interval |
| Deff | diffusion coefficient |
| WBC | White blood cell |
| NEUT | Neutrophil count |
| LYM | Lymphocyte count |
| MONO | Monocyte ratio |
| EO | Eosinophils |
| BASO | Basophil |
| RBC | Red blood cell count |
| HGB | hemoglobin |
| HCT | Hematocrit |
| MCV | Mean red blood cell volume |
| MCH | Mean erythrocyte Hb content |
| MCHC | Mean erythrocyte Hb concentration |
| RDW-CV | Erythrocyte volume distribution CV |
| PLT | Platelet count |
| MPV | Mean platelet volume |
| PDW | Platelet distribution width |
| TP | Total protein |
| ALB | Albumin |
| GLO | Globulin |
| ALT | Alanine aminotransferase |
| AST | Aspartate aminotransferase |
| GGT | γ-glutamyl transpeptidase |
| ALP | Alkaline phosphatase |
| TBIL | Total bilirubin |
| DBIL | Direct bilirubin |
| NBIL | Neutrophils bilirubin |
| TG. | Triglyceride |
| CHOL | Cholesterol |
| HDL-C | High density lipoprotein cholesterol |
| LDL-C | Low density lipoprotein cholesterol |
| APO-A1 | Apolipoprotein A1 |
| APO-B | Apolipoprotein B |
| GLU | Blood sugar |
| LDH | Lactic dehydrogenase |
| CK | Creatine kinase |
| CK-MB | Creatine kinase isoenzyme MB |
| UREA | Urea |
| UA | Uric acid |
| HCO3 | Bicarbonate |
| PHOS | Phosphate |
| OSM. | Plasma osmotic pressure |

**Table S1**. Pharmacokinetic parameters results of different administration groups. (n=6).

| rt-PA | | |  | _Sr_EcN_PL_ | | |
| --- | --- | --- | --- | --- | --- | --- |
| Parameter | Unit | Value |  | Parameter | Unit | Value |
| t1/2 | h | 0.06 |  | t1/2 | h | 19.92 |
| V | (mg)/(μg/ml) | 80.56 |  | V | (mg)/(μg/ml) | 306.24 |
| CL | (mg)/(μg/ml)/h | 831.01 |  | CL | (mg)/(μg/ml)/h | 10.65 |
| AUC 0-t | μg/ml*h | 0.99 |  | AUC 0-t | μg/ml*h | 62.85 |
| MRT | h | 0.09 |  | MRT | h | 28.74 |


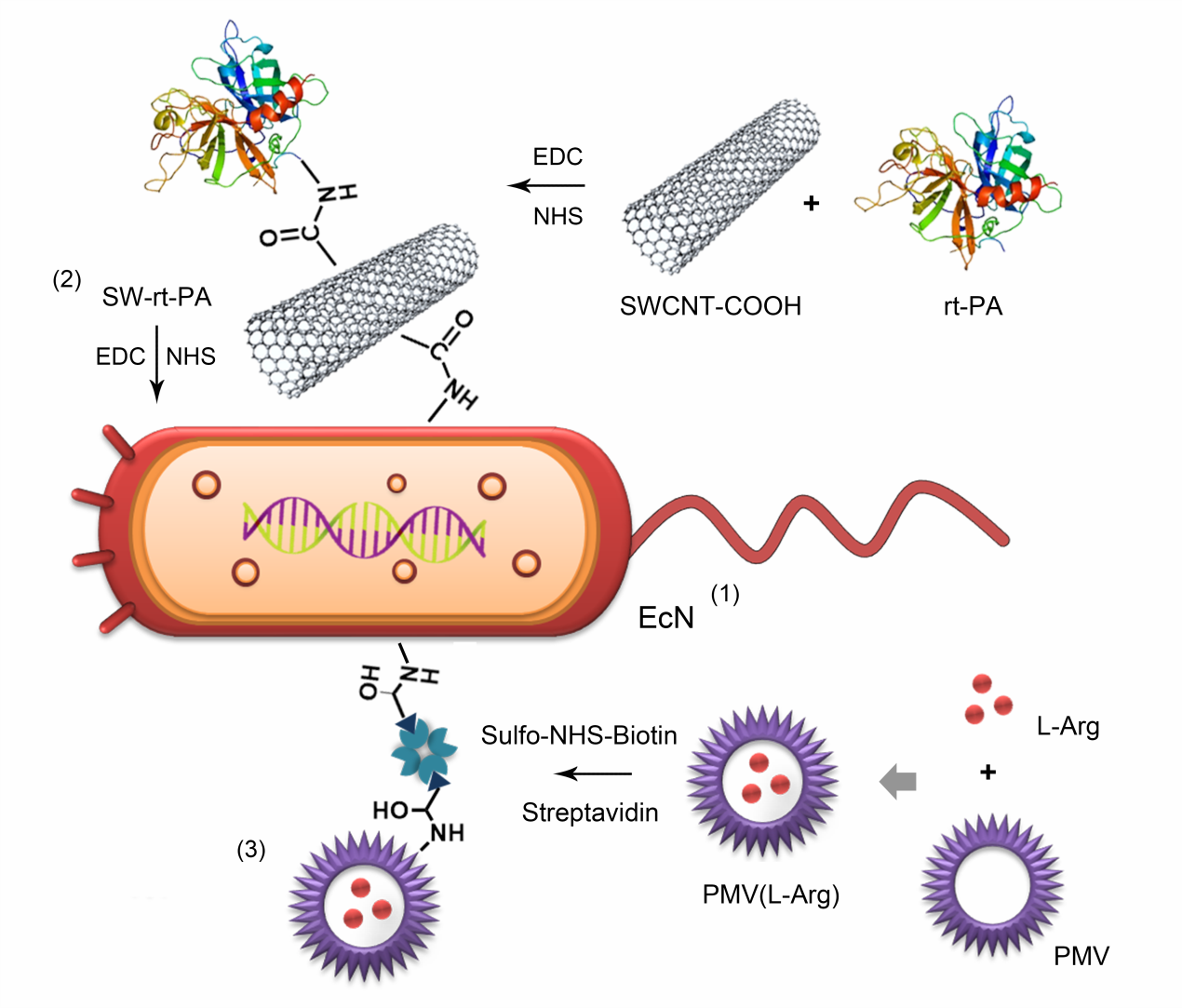


**Figure S1.** Synthesis methodology and mechanism of _Sr_EcN_PL_ micro-rod robots. (1) Cultivate EcN. (2) Modify SW-rt-PA on EcN to obtain _Sr_EcN. (3) Modify PMV(L-Arg) on _Sr_EcN to obtain _Sr_EcN_PL_.


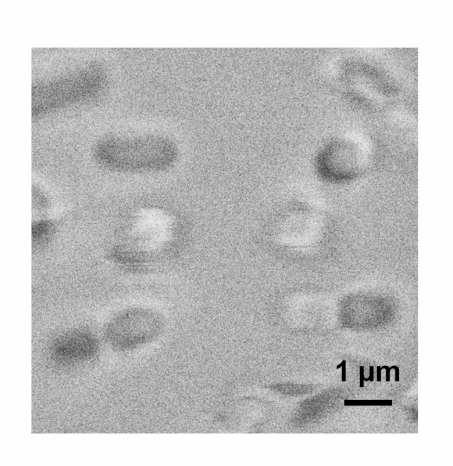


**Figure S2.** Laser scanning confocal microscopy with bright field analysis images of EcN.


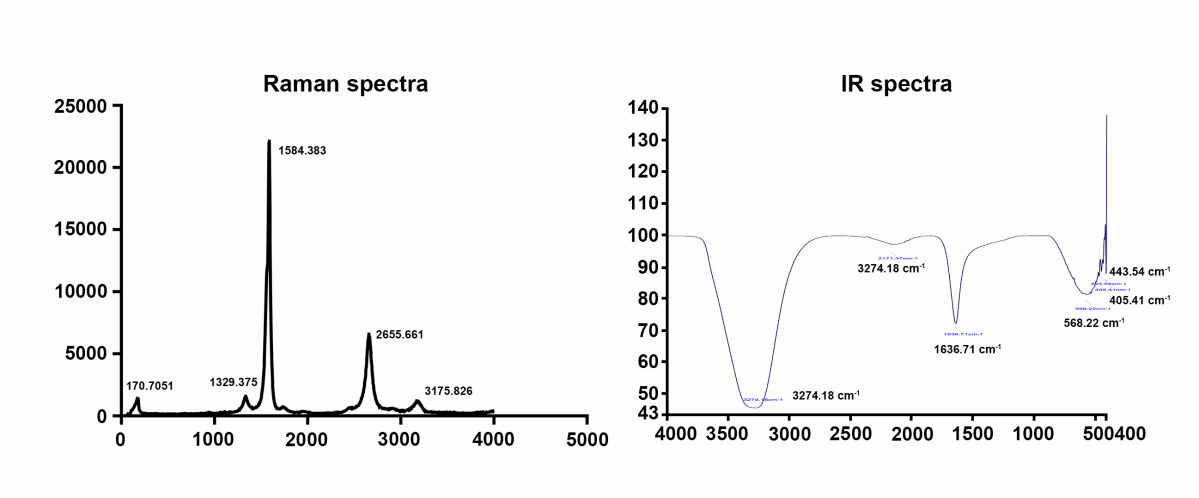


**Figure S3.** Raman spectra and IR spectra analyze results of SW-COOH.


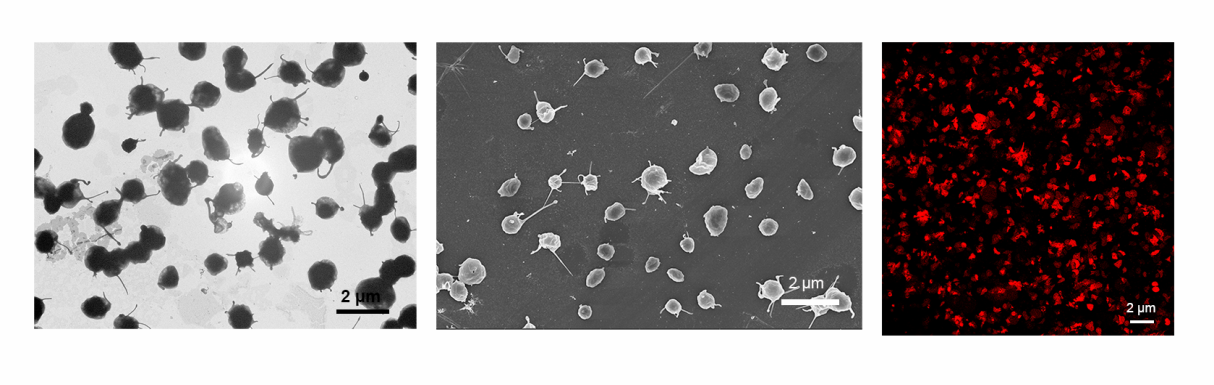


**Figure S4.** TEM, SEM and CLSM images of platelet extracted from mouse peripheral blood. The fluorescent probe DiR was used to label platelets.


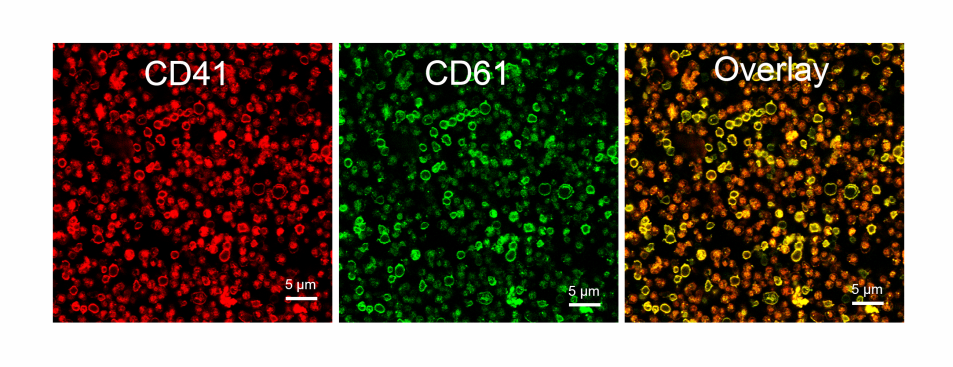


**Figure S5.** Immunofluorescence analysis of the characteristic proteins (CD61 and CD41) express on platelet surface.


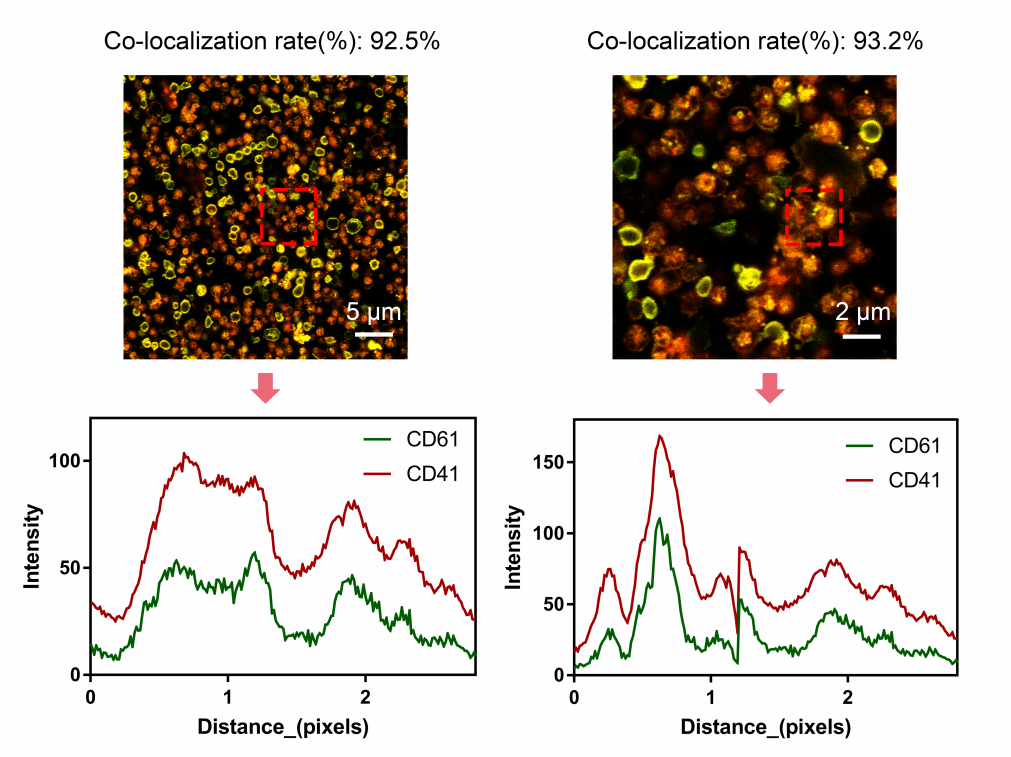


**Figure S6.** Fluorescence co-localization analysis of the characteristic proteins (CD61 and CD41) express on platelet surface.


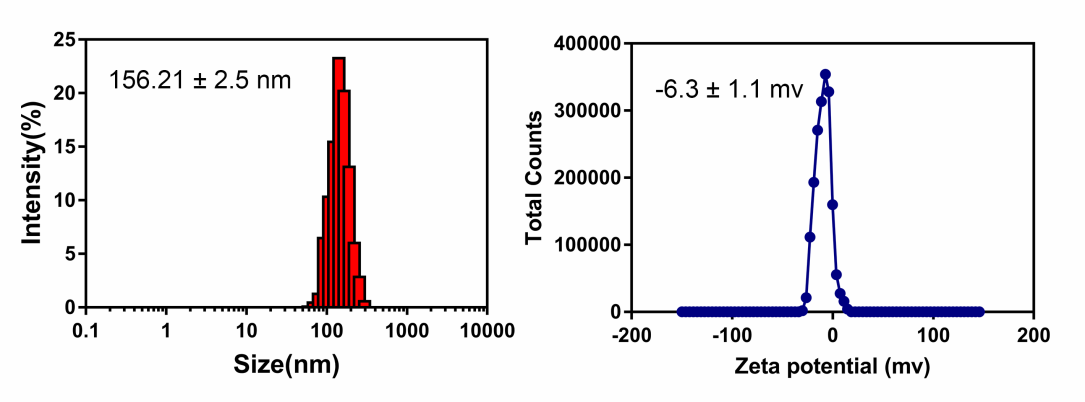


**Figure S7.** The particle size and zeta potential of PMV(L-Arg) nanoparticles.


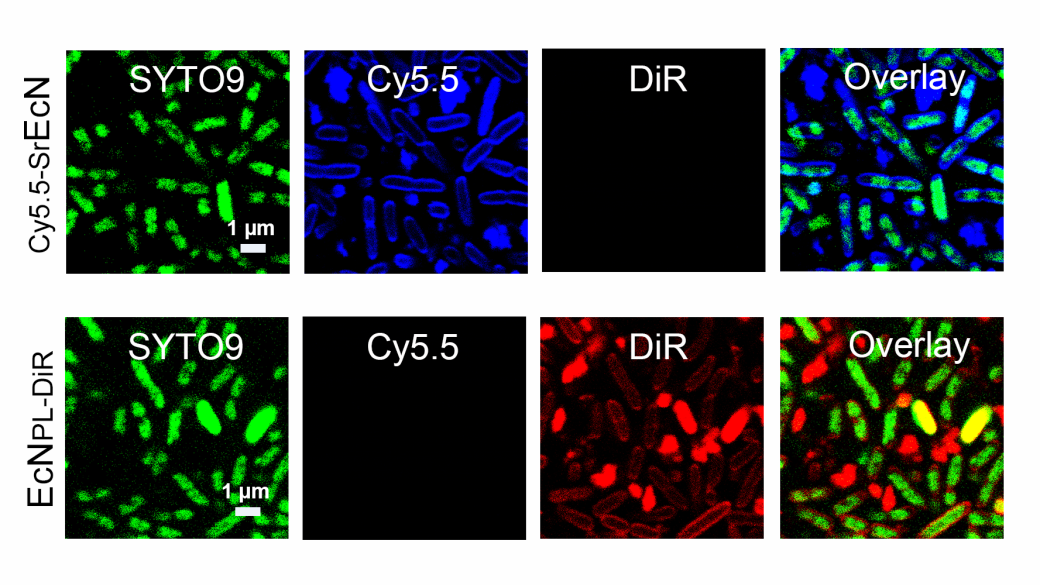


**Figure S8.** Laser confocal fluorescence imaging system was used to analysis of different micro-rod (EcN, _Sr_EcN and _Sr_EcN_PL_). Green fluorescence refers to probiotic labeled with probe SYTO9. Blue fluorescence refers to SW-rt-PA labeled with probe Cy5.5.


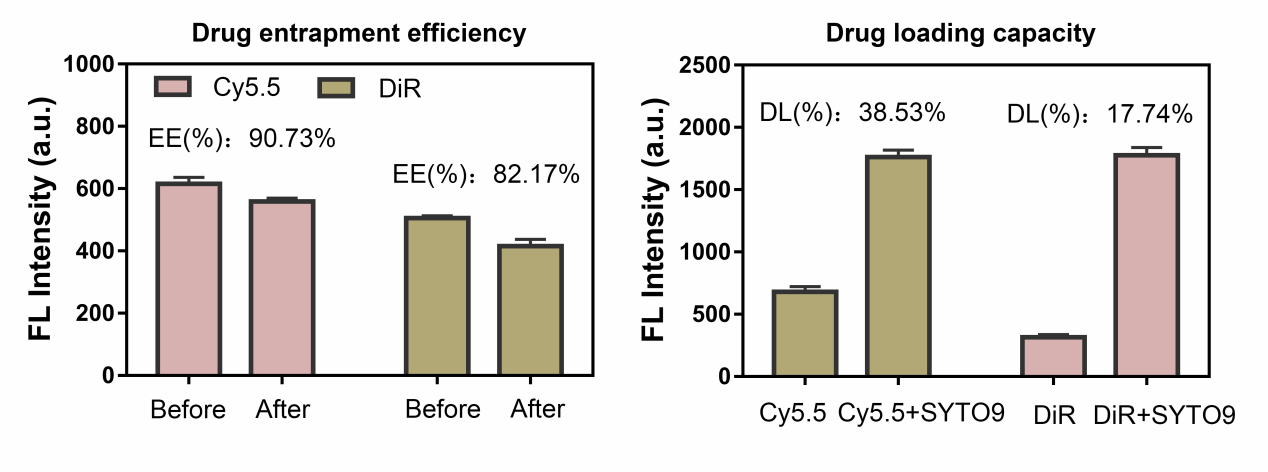


**Figure S9.** The drug entrapment efficiency and loading capacity of SW-rt-PA and PMV(L-Arg) in _Sr_EcN_PL_. (n = 3; mean ± SD).


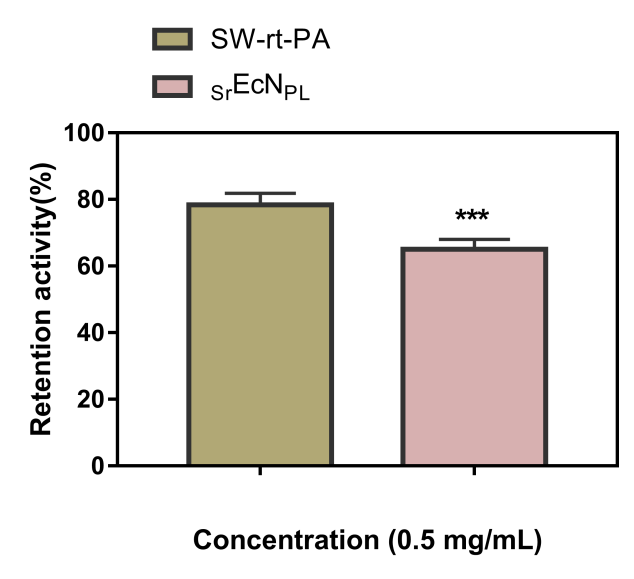


**Figure S10.** The retention activity of rt-PA in SW-rt-PA and _Sr_EcN_PL_ compared with the activity of corresponding concentration of free rt-PA (0.5 mg/mL) measured with the S-2288 substrate. (n = 3; mean ± SD). ***P < 0.001.


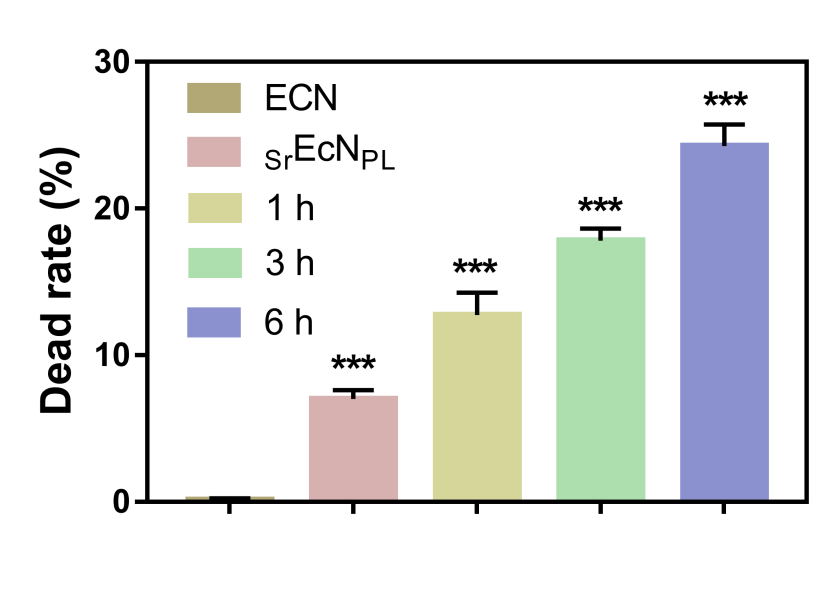


**Figure S11.** The dead rate analyze of different probiotic in different times. (n = 3; mean ± SD). ***P < 0.001.


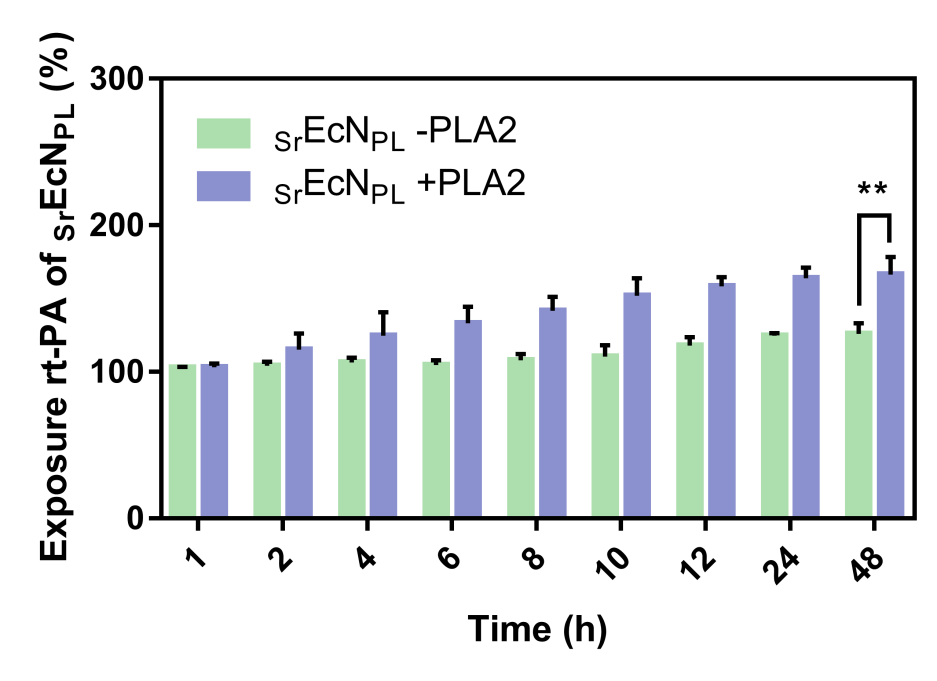


**Figure S12.** Exposure rate of rt-PA in _Sr_EcN_PL_ under different conditions (with or without PLA2). (n = 3; mean ± SD). **P < 0.01.


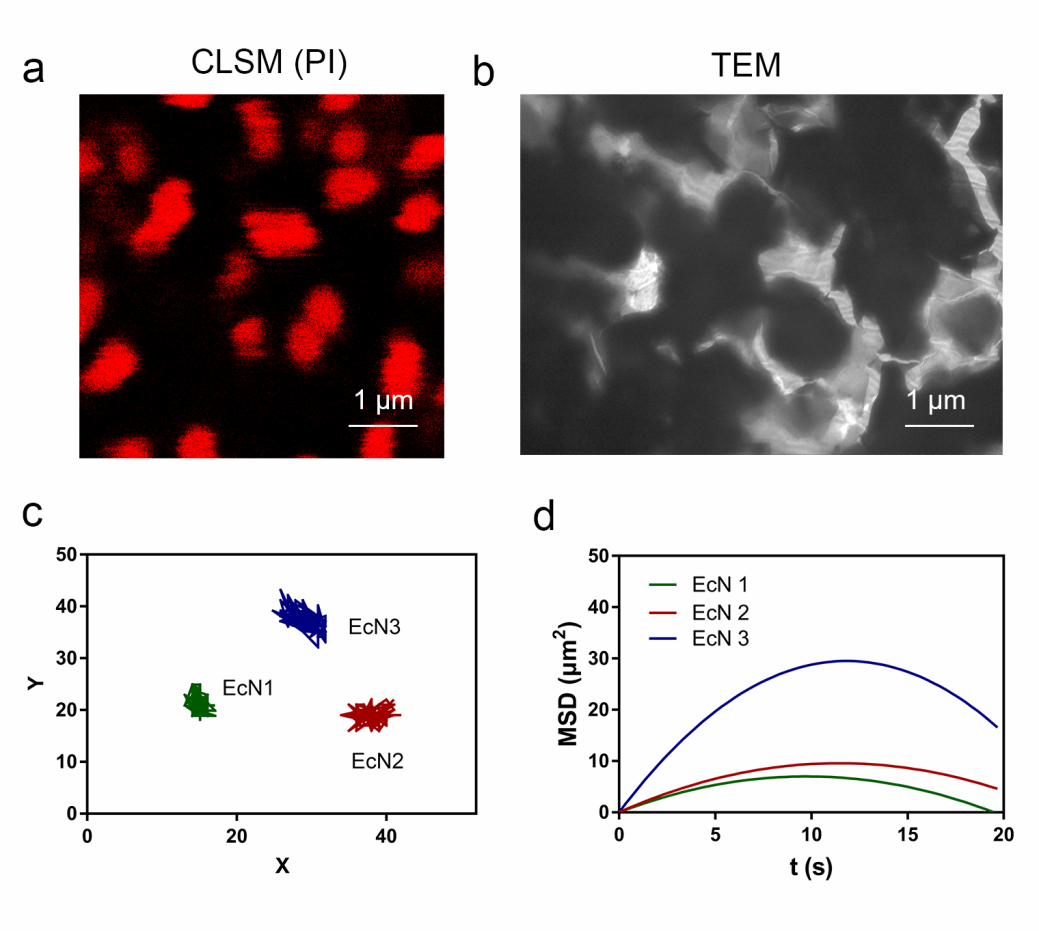


**Figure S13.** Evaluate of motion behavior of death probiotics. (a) The laser confocal fluorescence image of dead probiotics. (b) The TEM image of dead probiotics. (c) The motion trajectories of dead EcN labeled with probe PI in 20 s. (d) MSD of death probiotics.


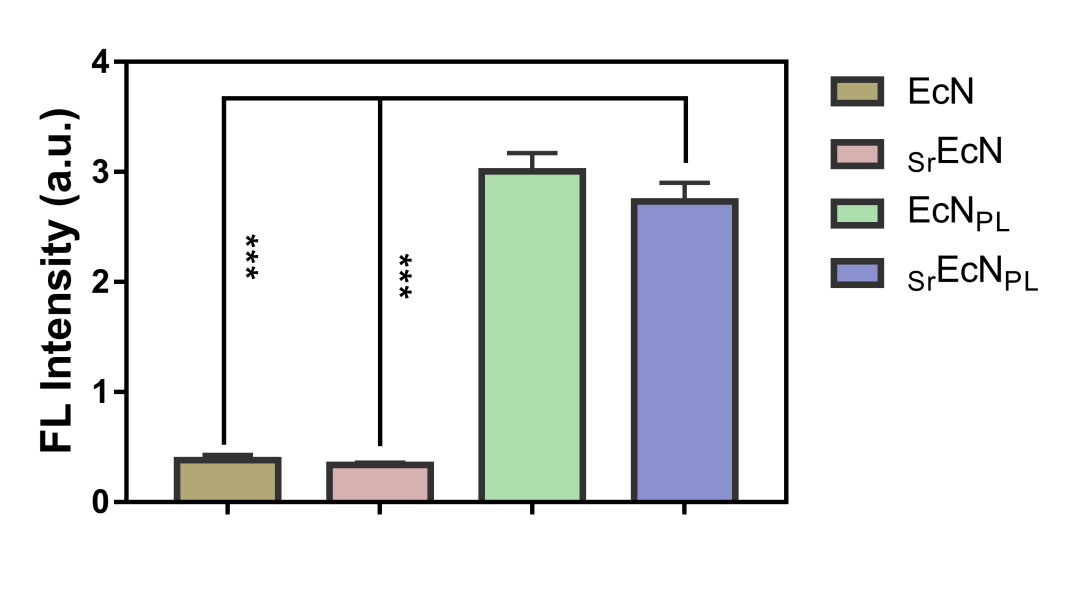


**Figure S14.** The fluorescence intensity analyze of SYTO9 in different groups. (n = 3; mean ± SD). ***P < 0.001.


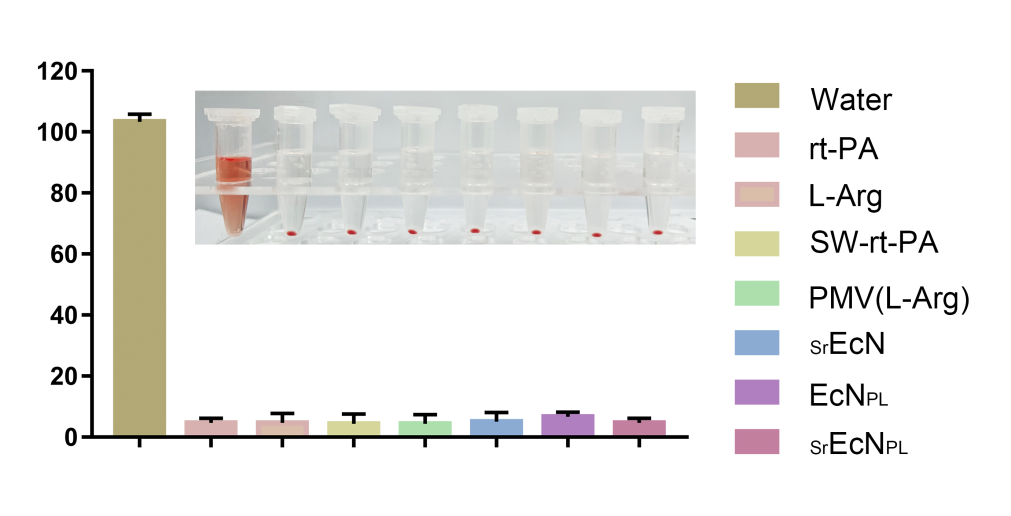


**Figure S15.** Hemolysis rate of red blood cells after incubated with different samples. (n = 3; mean ± SD).


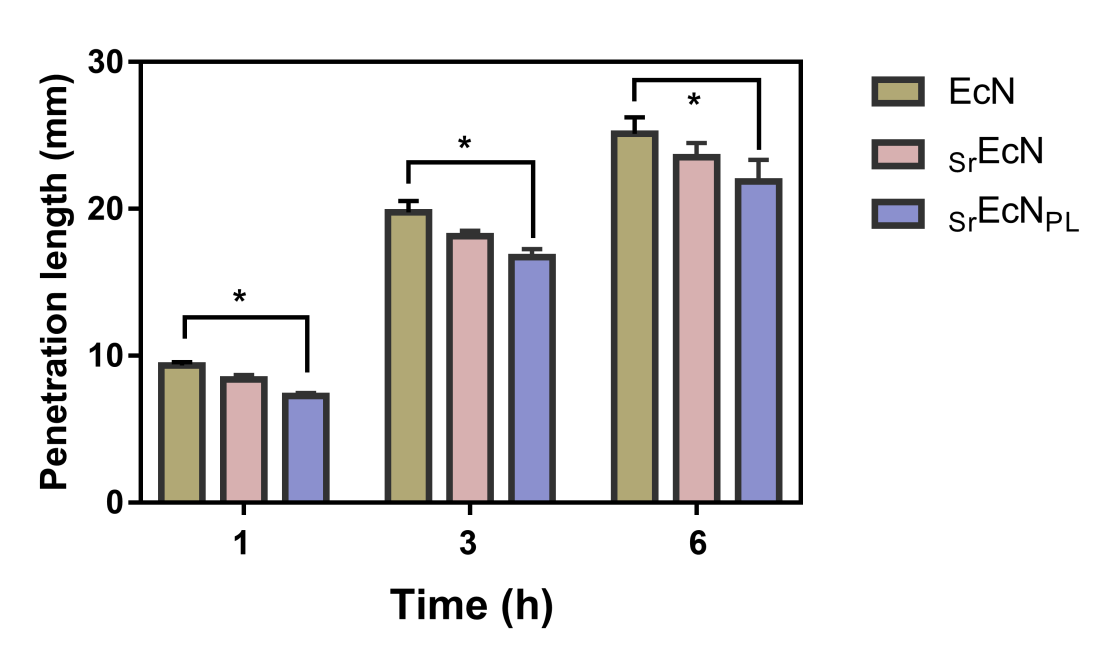


**Figure S16.** The thrombus blocks penetration length of different groups in different times. (n = 3; mean ± SD). *P < 0.05.


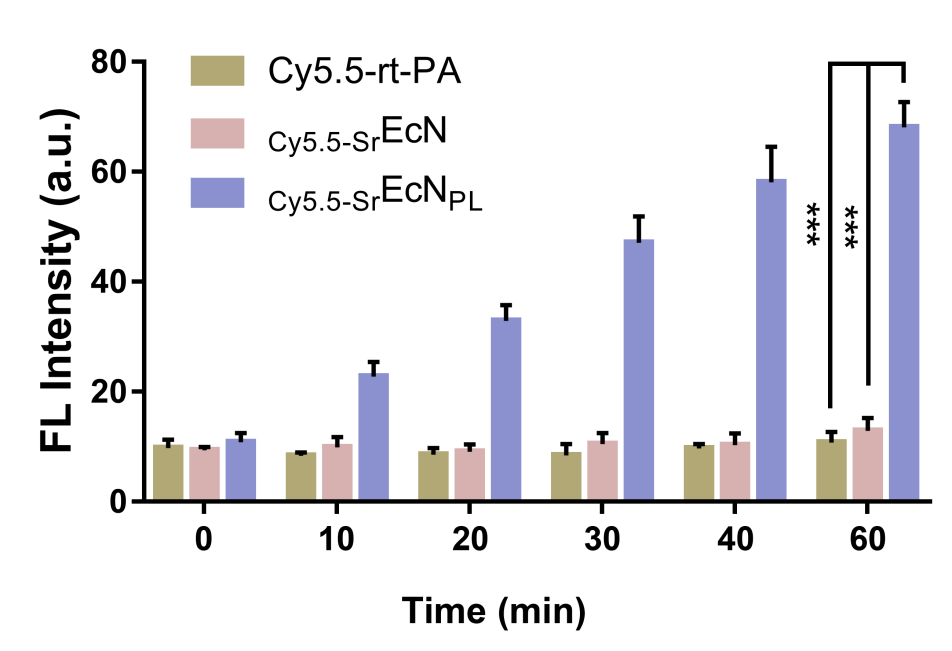


**Figure S17.** The fluorescence intensity analyze of LCCA blood vessels of different groups (Cy5.5-rt-PA, _Cy5.5-Sr_EcN, _Cy5.5-Sr_EcN_PL_) in different times. (n = 3; mean ± SD). ***P < 0.001.


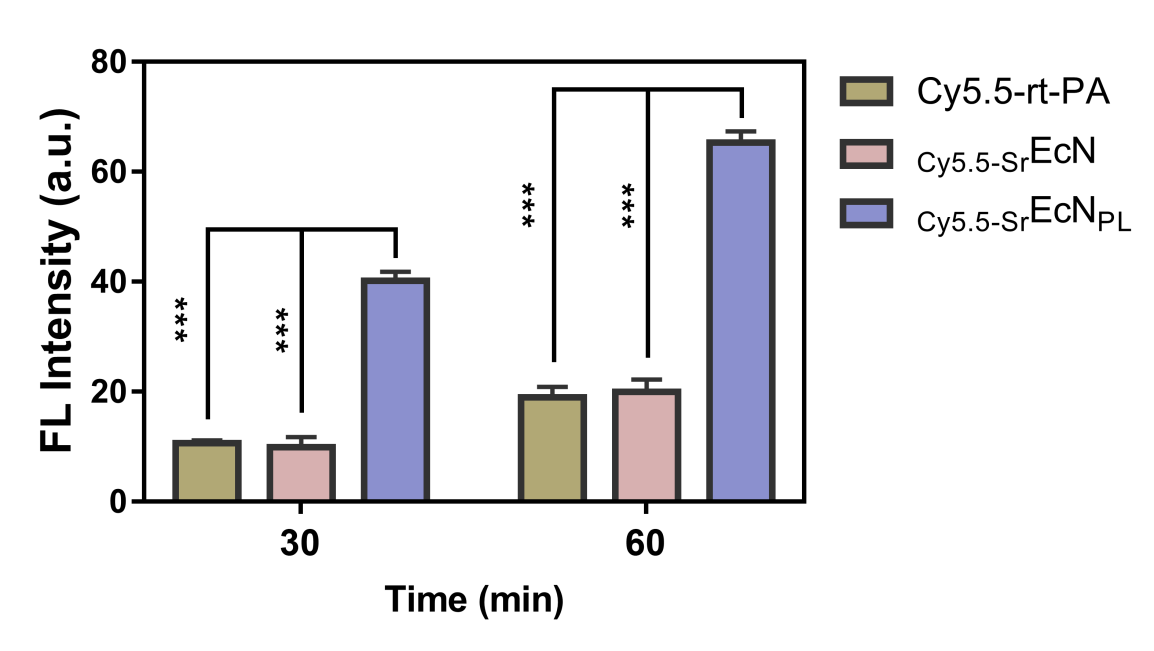


**Figure S18.** The fluorescence intensity analyze of LCCA blood vessel in different groups for different times. (n = 3; mean ± SD). ***P < 0.001.





**Figure S19.** Green Fluorescence gray scale images of LCCA blood vessels from different groups (Cy5.5-rt-PA, _Cy5.5-Sr_EcN, _and Cy5.5-Sr_EcN_PL_) in different times. Scale bars, 100 µm.


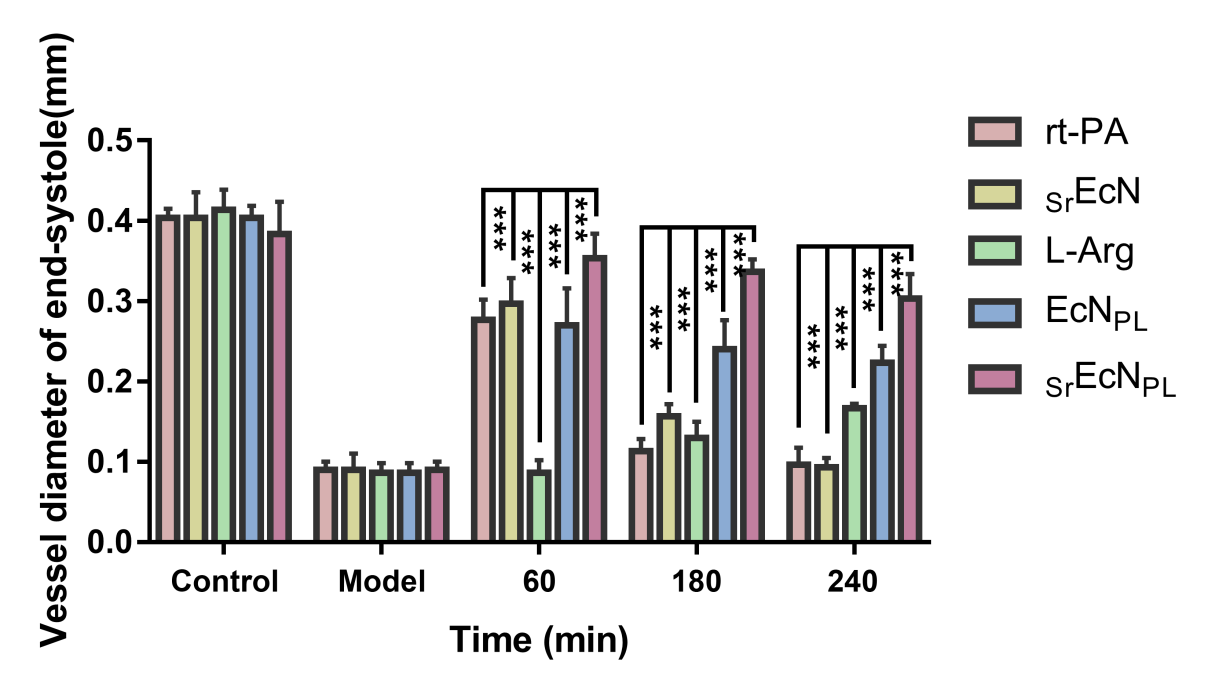


**Figure S20.** Fluorescence intensity analyze of LCCA blood vessels from different groups (saline, L-Arg, EcN_PL_, _Sr_EcN_PL_, 1.5×10^4^ CFU/mL, 0.4 mg rt-PA) treated for 3 h and stained with DAF-FM DA probe. (n = 3; mean ± SD). ***P < 0.001.


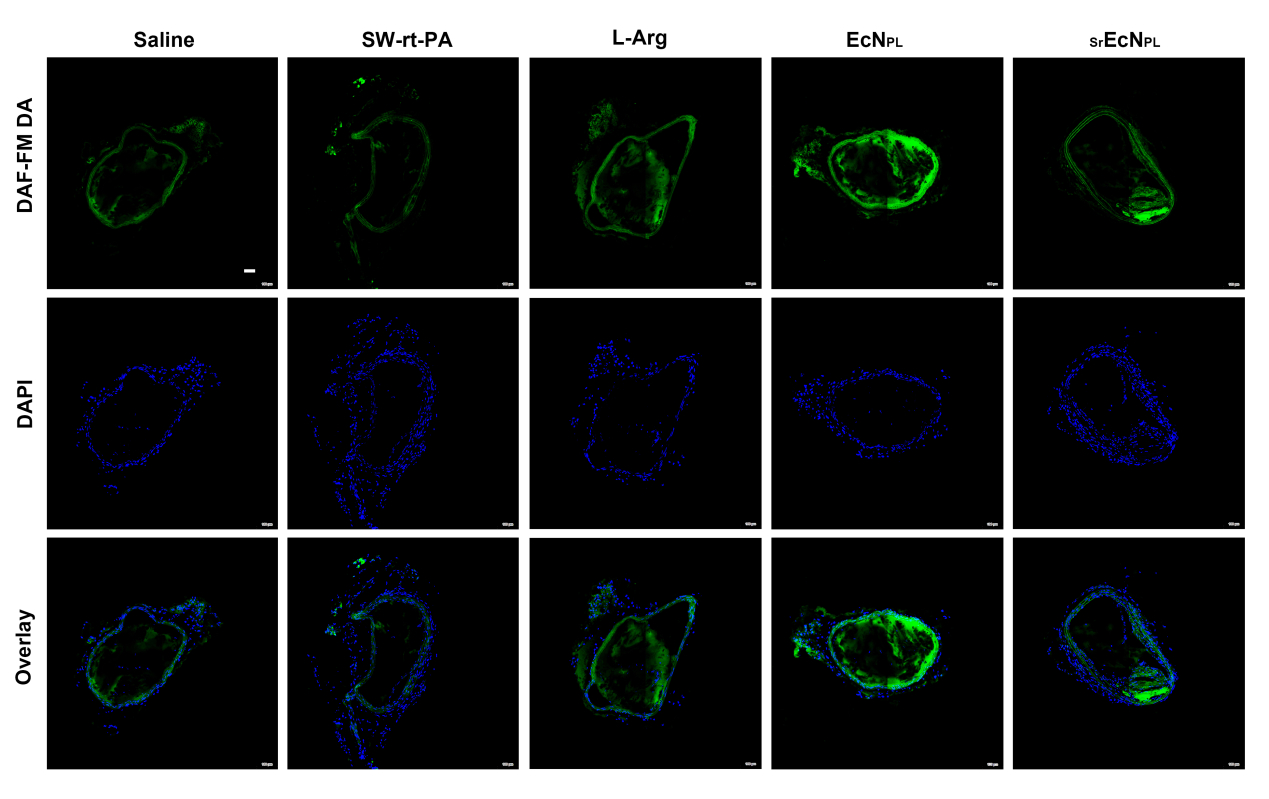


**Figure S21.** Fluorescence images of LCCA blood vessels from different groups (saline, L-Arg, EcN_PL_, _Sr_EcN_PL_, 1.5×10^4^ CFU/mL, 0.4 mg rt-PA) treatment for 3 h, and stained with DAF-FM DA probe. Scale bars, 100 µm.


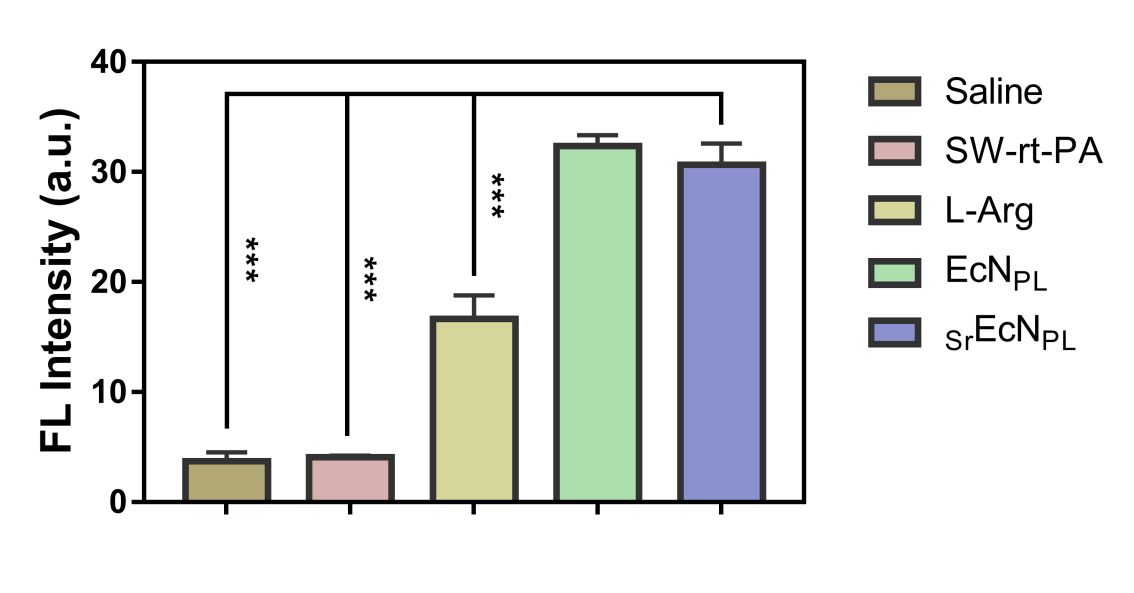


**Figure S22.** Fluorescence intensity analyze of LCCA blood vessels from different groups (saline, L-Arg, EcN_PL_, _Sr_EcN_PL_, 1.5×10^4^ CFU/mL, 0.4 mg rt-PA) treated for 3 h and stained with DAF-FM DA probe. (n = 3; mean ± SD). ***P < 0.001.


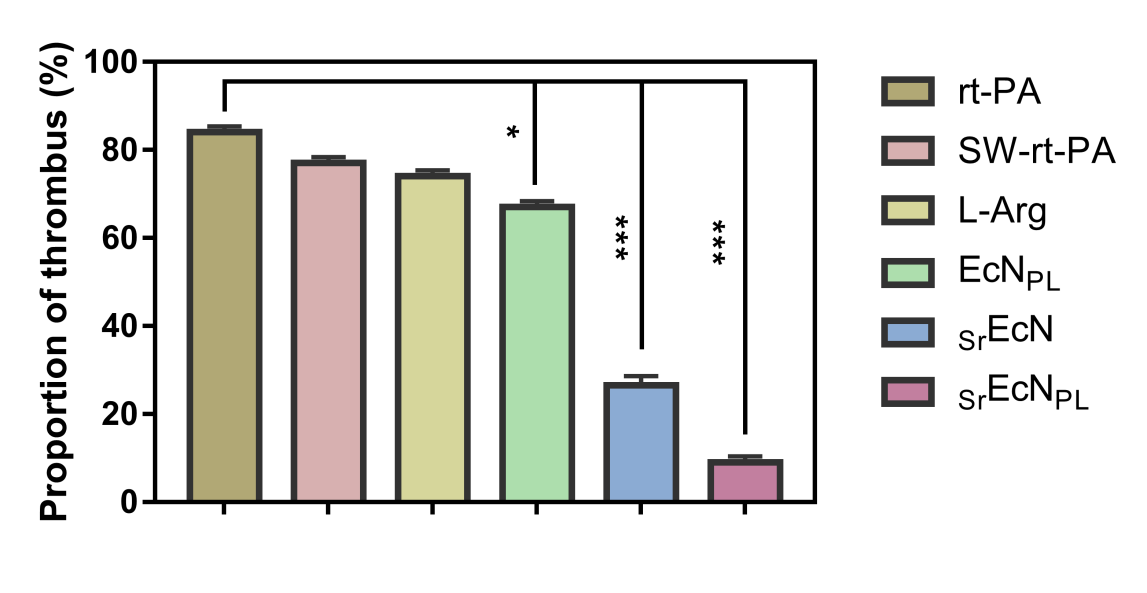


**Figure S23.** The average thrombus proportion of H&E and Masson staining of blood vessels from different treatment groups (rt-PA, SW-rt-PA, L-Arg, EcN_PL_, _Sr_EcN, _Sr_EcN_PL_, 1.5×10^4^ CFU/mL, 0.4 mg rt-PA) for 4 h. (n = 3; mean ± SD). *P < 0.05, ***P < 0.001.


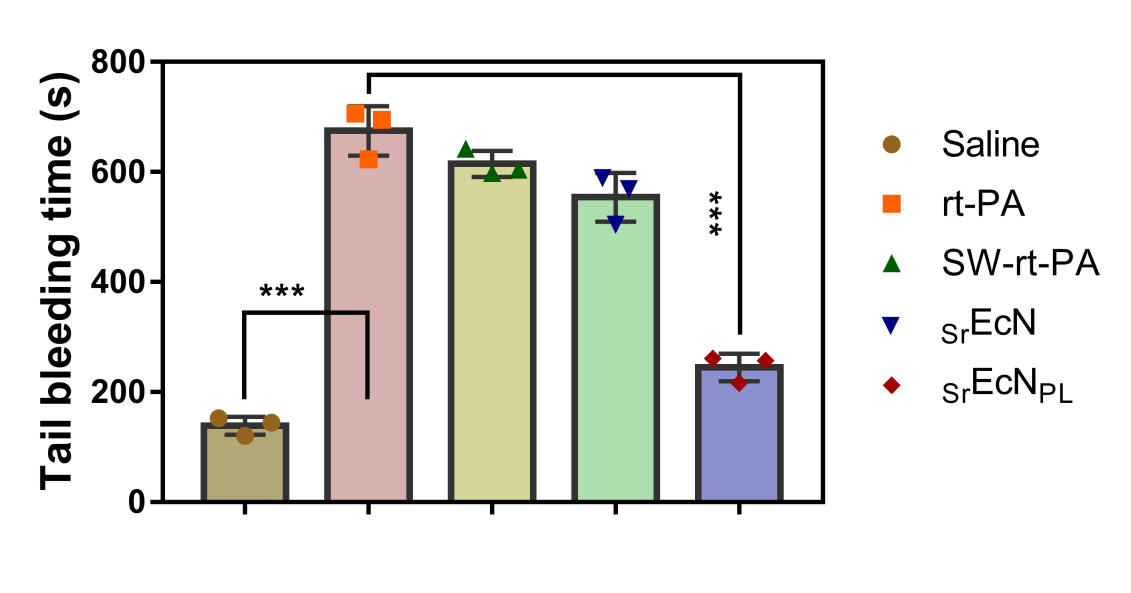


**Figure S24.** The hemorrhagic time of the mice treated with different samples. (n = 3; mean ± SD). ***P < 0.001.


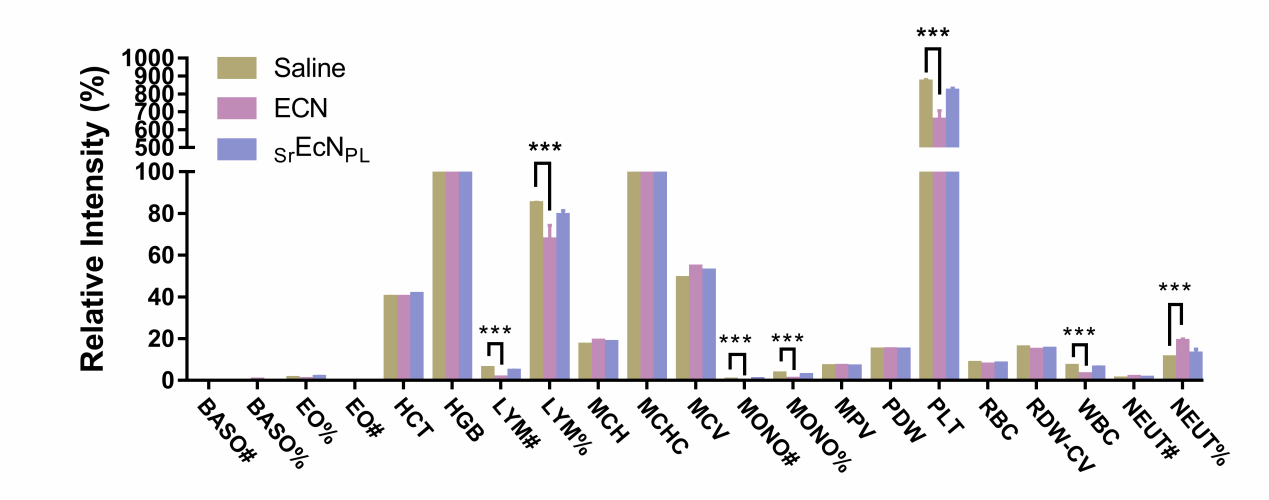


**Figure S25.** Blood routine data analyze of different administrate groups. (n = 3; mean ± SD). ***P < 0.001.


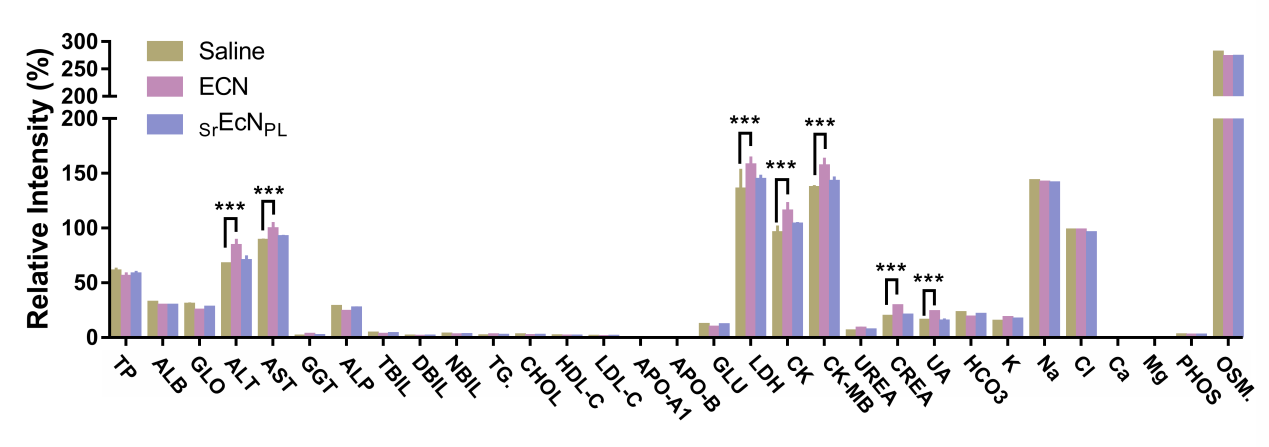


**Figure S26.** Blood biochemical data analyze of different administrate groups. (n = 3; mean ± SD). ***P < 0.001.


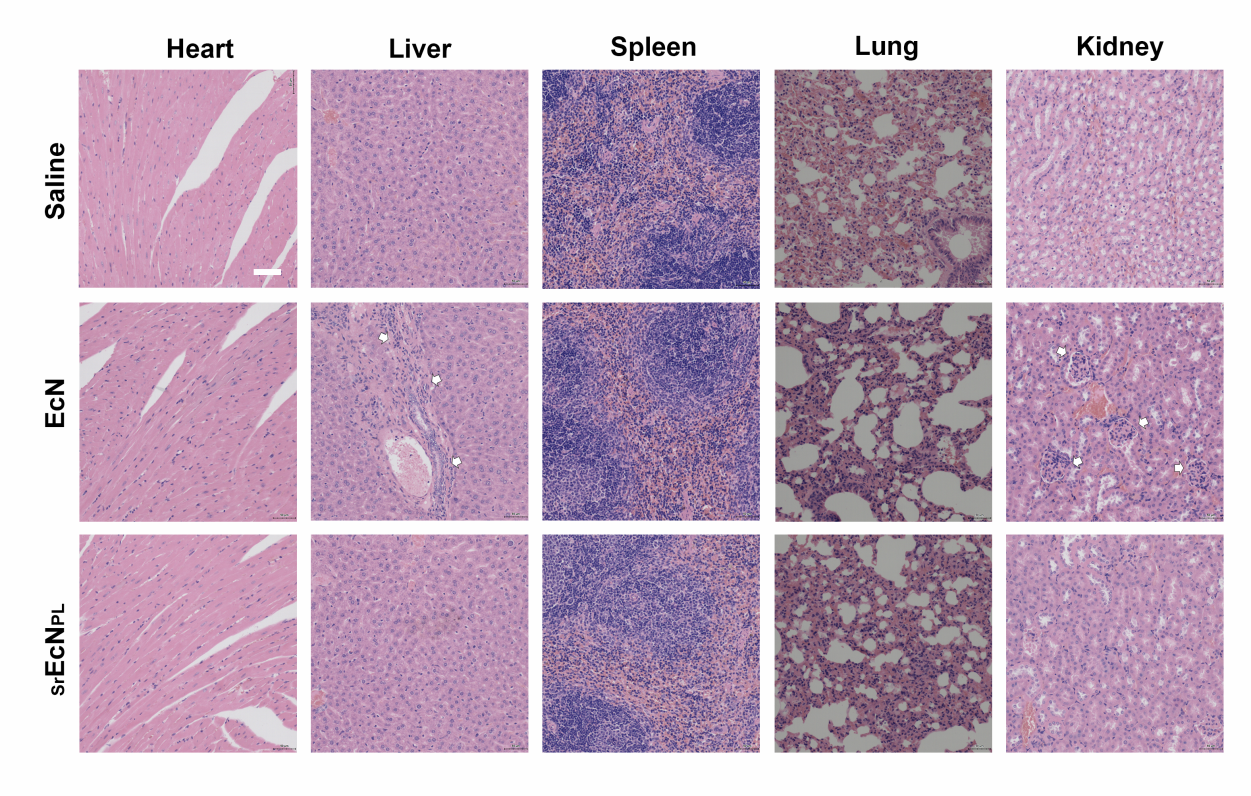


**Figure S27.** H&E stain analyze of five organs from mice treated by different administration groups. Scale bar, 100 µm. The white arrows refer to the site of inflammatory cell infiltration.
